# Evolutionary Consequences of Functional and Regulatory Divergence of HD-Zip I Transcription Factors as a Source of Diversity in Protein Interaction Networks in Plants

**DOI:** 10.1007/s00239-023-10121-4

**Published:** 2023-06-23

**Authors:** Natalia Żyła, Danuta Babula-Skowrońska

**Affiliations:** Institute of Plant Genetics, Polish Academy of Sciences, Strzeszyńska 34, 60-479 Poznan, Poland

**Keywords:** Transcription factors, HD-Zip I subfamily, Functional diversity, Whole genome duplication, Plant growth and development

## Abstract

The HD superfamily has been studied in detail for several decades. The plant-specific HD-Zip I subfamily attracts the most attention because of its involvement in plant development and stress responses. In this review, we provide a comprehensive insight into the evolutionary events responsible for the functional redundancy and diversification of the HD-Zip I genes in regulating various biological processes. We summarized the evolutionary history of the HD-Zip family, highlighting the important role of WGDs in its expansion and divergence of retained duplicates in the genome. To determine the relationship between the evolutionary origin and functional conservation of HD-Zip I in different species, we performed a phylogenetic analysis, compared their expression profiles in different tissues and under stress and traced the role of orthologs and paralogs in regulating developmental processes. We found that HD-Zip I from different species have similar gene structures with a highly conserved HD and Zip, bind to the same DNA sequences and are involved in similar biological processes. However, they exhibit a functional diversity, which is manifested in altered expression patterns. Some of them are involved in the regulation of species-specific leaf morphology and phenotypes. Here, we discuss the role of changes in functional domains involved in DNA binding and protein interaction of HD-Zip I and in *cis*-regulated regions of its target genes in promoting adaptive innovations through the formation of *de novo* regulatory systems. Understanding the role of the HD-Zip I subfamily in organism-environment interactions remains a challenge for evolutionary developmental biology (evo-devo).

**Supplementary Information:**

The online version contains supplementary material available at 10.1007/s00239-023-10121-4.

## Introduction

Regulation of gene transcription is a complex process in which specific proteins act as commutators within regulatory cascades controlling the functions of genes. Transcription-associated proteins are divided into three functional groups: DNA-binding transcription factors (TFs), transcription cofactors and proteins associated with chromatin structure. TFs are protein-coding genes that recognize and bind to specific DNA sequences in promoters of target genes to control biological processes in all living organisms. Their activity can be modulated by mechanisms involving their synthesis, subcellular localization, interaction with other proteins and posttranslational modifications.

In plants, the evolutionary history of TFs is clearly associated with dramatic geological and climatic changes during land colonization that required the developing of new regulatory mechanisms. The rapid adaptation of plants to unfavorable environmental conditions such as increased CO_2_ concentration, light intensity, desiccation and limited nutrient availability is attributed to the increase in the number of genes that resulted from whole genome duplications (WGDs) and then their gradual sub- or neofunctionalization, leading to morphological, physiological and regulatory changes in organisms (Rensing 2014). Most copies of TFs were retained in genomes after the α-WGD event because regulatory proteins are protected from mutations due to their pleiotropic effects (Prud’homme et al. 2007). However, a gene duplicate is often released from negative selective pressure and even single-nucleotide mutations in the DNA-binding or interaction domains can alter its binding preferences and promote gene regulatory network (GRN) evolution (Paris et al. 2013; Rosas et al. 2014). In particular, the retention and subsequent diversification of duplicated TFs involved in stress responses and affecting to changes in stress signaling pathways in plants have been documented (Mizoi et al. 2012; Yang et al. 2014). Some studies suggest that phenotypic diversity is due to mutations in the *cis*-regulatory motif rather than in the coding sequence of TF (Wray 2007). A genome-wide analysis of *cis*-regulatory sites in paralogs of *A. thaliana* confirmed that changes in their architecture are reflected in changes in the regulatory network (Arsovski et al. 2015). In contrast, regions outside DNA-binding domains involved in protein–protein interactions are conserved, especially for hormone-related TFs such as ARF, PIF, MYC2, FHY1 and ABI3A (Romani, Moreno 2021). Therefore, further studies are needed to determine the mechanisms underlying the conservatism or functional divergence of DNA binding preference between WGD-derived members of TF families and their target genes.

The abundance of TFs has been confirmed in many plants whose genomes have been sequenced and they account for between 0.5 and 13% of all protein-coding genes (PlantTFDB v5.0 http://planttfdb.gao-lab.org/; Table S1). These regulatory genes were classified according to the specificity of their DNA-binding domain, from 46 families in *Trifolium pratense* and *Helianthus annuus* to 71 families in *Kalanchoe laxiflora* (Luscombe et al. 2000; Table S1). The observed differences in gene abundance among plant species are due to their polyploidy status or genome duplication. The *A*. *thaliana* genome encodes 2296 TFs classified into 58 families according to specific DNA-binding domains, structural features and functions (PlantTFDB v5.0; http://planttfdb.gao-lab.org/, Table S1). Approximately 60% of them are from three rounds of WGD: γ (1R), β (2R), and α (3R) (Maere et al. 2005). Thus, this number is 1.3 and 1.7 times higher than in *Drosophila* and *Caenorhabditis elegans*, respectively, and is consistent with the eukaryotic evolutionary model, which indicates a higher rate of gene expansion in land plants than in animals (Shiu et al. 2005). About 40% of *A. thaliana* TFs such as HD-Zip, AP2/EREBP, ARF, NAC, SBP, GARP, MADS-box, ABI3/VP1 and WRKY are plant-specific TFs. This confirms an independent evolution of lineage-specific TF families that began as early as the Precambrian (Catarino et al. 2016; Bowman et al. 2017; Wilhelmsson et al. 2017; Rensing 2014; Johnson 2017).

The homeobox proteins are transcription factors found in all living organisms that contain the highly conserved homeodomain (HD) involved in DNA-binding. The first plant HD-containing gene was discovered in maize by Vollbrecht and coworkers (1991). The *KNOTTED1* gene was identified by transposon tagging and its constitutive expression in transgenic maize plants showed knot-like structures in the leaves. In plants, the superfamily of HD genes is divided into 11 families: BEL, DDT, HD-Zip, KNOX, LD, NDX, PHD, PINTOX, PLINC, SAWADEE and WOX (Mukherjee et al. 2009). The plant-specific HD-Zip gene family is characterized by the presence of the highly conserved 60 amino acid HD adjacent to the less conserved leucine zipper motif (LZ) (Ariel et al. 2007). It is divided into four subfamilies: HD-Zip I, HD-Zip II, HD-Zip III and HD-Zip IV, depending on DNA-binding specificity, gene structure, number of heptad repeats in LZ, presence of additional motifs and biological functions. The HD is crucial for recognition and binding of these transcription factors to the *cis*-regulatory element in target genes, whereas LZ has some heptad repeats with a leucine residues in the center position and functions as a dimerization motif that is critical for efficient DNA binding (Tron et al. 2004). The HD-Zip proteins interact with DNA only as dimers. The three-dimensional structure of HD contains three characteristic α-helices, of which the second and third helices form a helix-turn-helix DNA-binding motif (Desplan et al. 1988). The *HD-Zip I* and *HD-Zip II* genes recognize the pseudopalindromic sequence CAATNATTG with the central nucleotides A/T and C/G, respectively, in the regulatory regions of target genes (Sessa et al. 1993; Palena et al. 1999). Whereas, the *HD-Zip III* and *HD-Zip IV* genes recognize GTAAT(G/C)ATTAC and CATT(A/T)AATG sequences, respectively, in their target genes (Sessa et al. 1998; Tron et al. 2001).

In this review, we provide new insights into the evolution and evolutionary processes that led to the functional redundancy and/or diversification of the HD-Zip I transcription factors in plants. We summarized the evolutionary history of the HD-Zip family, highlighting the important role of WGDs in the separation of four subfamilies in flowering plants and the functional diversity of the retained duplicates in the genome. To correlate the common evolutionary origin and functional conservation of orthologous and paralogous genes, we defined the phylogenetic relationships of the 483 HD-Zip I proteins from different species (including monocots, dicots, ferns and mosses) based on amino acid sequence similarity. We then compared their expression profiles in different tissues and under stress conditions. We also tracked the role of orthologous genes from different species and paralogous genes from the same species in regulating growth and development and in responding to stress. These analyzes confirmed the functional complexity of the HD-Zip I subfamily, resulting from its evolution in the context of WGD and the adaptation of different species to a rapidly changing environment. Many of these genes have similar structures, bind to the same DNA sequences in target genes and exhibit similar expression profiles during developmental processes and under stress, confirming their partial functional redundancy. However, some of them exhibit a functional diversity, manifested in altered expression patterns and related to the site-specificity of their activity (organs or tissues) or to external stimuli such as water availability and light. In addition, some members of HD-Zip I are involved in the regulation of species-specific leaf morphology and phenotypes. We discuss the role of changes in functional domains involved in DNA binding and protein interaction of HD-Zip I proteins and in *cis*-regulated regions of their target genes, which are responsible for the evolution of morphological diversity in plants and promote adaptive innovations through the formation of de novo regulatory systems to control various biological processes. Understanding the role of the HD-Zip I subfamily in organism-environment interactions remains a challenge for evolutionary developmental biology (evo-devo).

## Evolution of the Homeodomain Leucine Zipper (HD-Zip) Gene Family in Plants

**Fig. 1:**
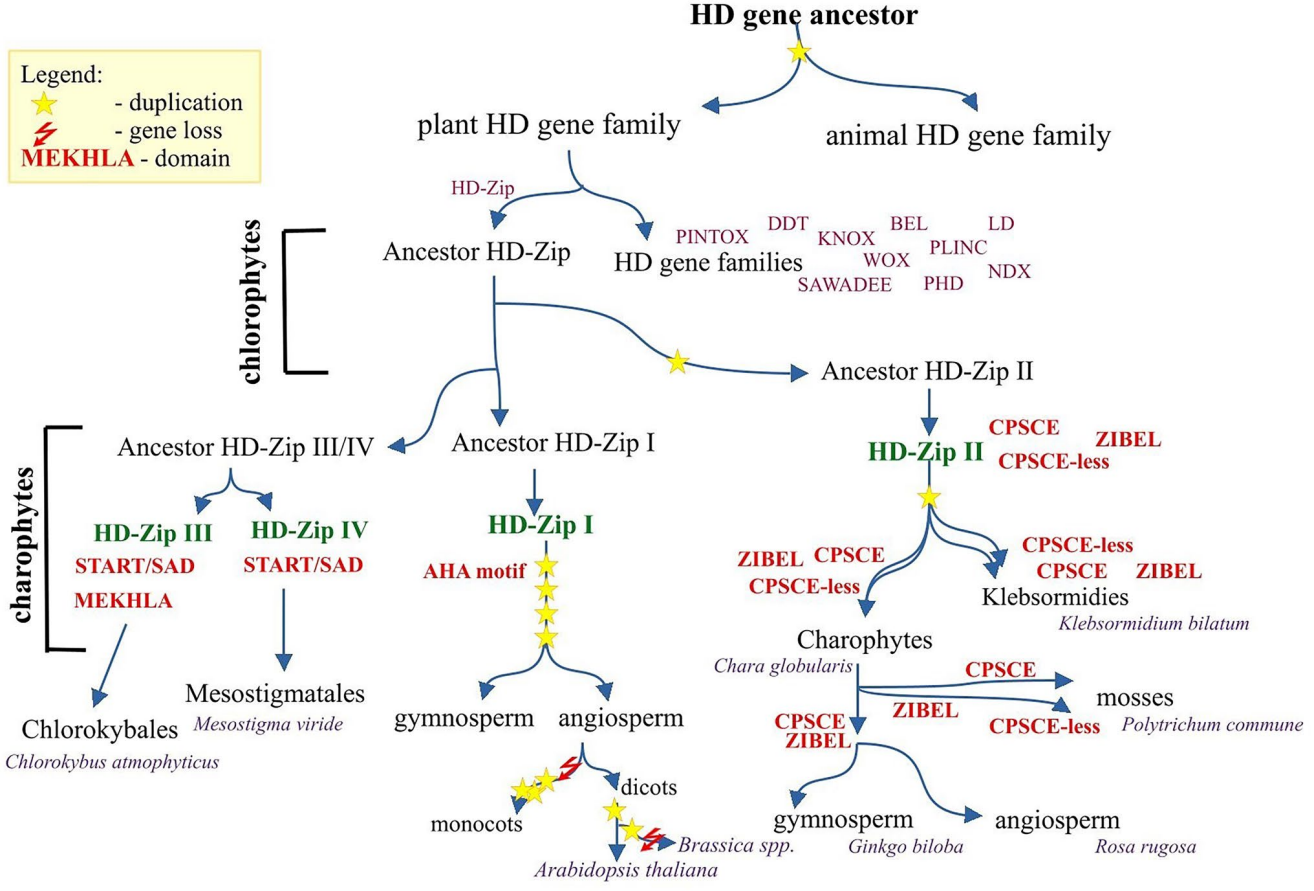
The evolutionary model of the HD-Zip gene family in plants. Major events including duplications (marked by asterisks) or gene loss (marked by lightning), which took place at different stages of the plant evolution, have been shown. The additional domains are colored in red (Color figure online)

Together with the availability of fully sequenced genomes, our knowledge of the evolution of the HD superfamily has increased significantly. Initial alignments of HD sequences from different taxa showed a closer evolutionary relationship between plants and animals than between different plant families (Bharathan et al. 1997). This suggests that the divergence of HDs began before the separation of plants, animals and fungi, and only later within the plant kingdom. In plants, almost all HD families except LD and NDX have already been identified in chlorophytes (Romani et al. 2018). Members of the LD family were found in charophytes and the NDX family only in land plants, suggesting that these families were lost in chlorophytes due to gene loss or arose later in evolution. Among the *HD* superfamily, the *HD-Zip* family is one of the best characterized gene groups in plants. The numerous phylogenetic studies have shed light on the complex evolution of the four classes in this family (Prigge and Clark 2006; Mukherjee et al. 2009; Romani et al. 2018). However, little is known about its origin, especially in the early stages of plant differentiation. Therefore, it is controversial whether the HD-Zip family arose as a new lineage within the HD gene superfamily or whether it was formed by fusion of protein domains (Schena and Davis 1992). The evolutionary model of the HD-Zip gene family assumes that a single HD-Zip-like protein arose during early chlorophyte evolution and then gradually diverged into the four classes in charophytes (Fig. 1). The HD-Zip II-like protein first evolved in chlorophytes and was later inherited by streptophytes. Phylogenetic analysis of these proteins in different plant taxa revealed two clades with presence or absence of the specific CPSCE sequence in their C-terminal part (Romani et al. 2018). These two clades have a common origin and underwent the duplication event before the divergence of Klebsormidiales. However, the clade with the CPSCE-less sequence is conserved only in charophytes and mosses and has been lost in vascular plants. This evolutionary fate of two lineages of HD-Zip II was further supported by differences in the intron–exon structure of genes with CPSCE-less and CPSCE motifs. In addition, the ZIBEL-like motif was detected at the N-terminus of HD-Zip II proteins in Klebsormidiales and is also conserved in all land plants. According to the phylogenetic tree, HD-Zip I, HD-Zip III and HD-Zip IV are monophyletic groups in land plants (Romani et al. 2018). The HD-Zip III- and HD-Zip IV-like proteins evolved from a common ancestor during the early evolution of streptophytes but before the divergence of *Klebsormidium*. In parallel, the STeroidogenic Acute Regulatory protein-related lipid Transfer/START-Associated Domain (START/SAD) motifs were incorporated into the C-terminal end of the HD-Zip III and HD-Zip IV proteins. The methionine-glutamine-lysine-histidine-leucine-alanine (MEKHLA) motif was found only in the HD-Zip III proteins, appeared in the *Klebsormidium* species and was retained in later stages of evolution. Although the *HD-Zip III* and *HD-Zip IV* genes have a common ancestor, they showed different evolution of exon–intron structure. The gene structure of the members of *HD-Zip IV* with about 10 exons is conserved among plant taxa, while *HD-Zip III* underwent modifications that resulted in changes in the number of their exons increasing from 13 in *Klebsormidium nitens* to 18 in *Marchantia* and *Arabidopsis*. In contrast, the phylogenetic analyzes of HD-Zip I-like proteins in different plant taxa were unable to clearly determine their evolutionary fate because of divergent sequences. This is probably due to fact that they evolved faster than the HD-Zip II proteins in both monocotyledons and dicotyledons (Sakakibara et al. 2001). The HD-Zip I genes eventually evolved in Klebsormidiophyceae, which is confirmed by the specific gene structure in Klebsormidiales with an intron within the HD domain that was not found in *Marchantia* and other land plants. Some HD-Zip I proteins have the AHA motif at the C-terminus, which probably occurred before the divergence of Charales and is conserved in other charophytes. In land plants, this AHA motif is conserved in bryophytes, but in some angiosperm lineages it degenerated later. The HD-Zip I proteins of the mosses also share other regulatory motifs in their N-and C-terminal regions. All this suggests a low level of diversification of HD-Zip I in *Physcomitrella patens* with functional redundancy.

In the evolution of the HD-Zip family, at least some duplication events have been confirmed to have had an impact on its expansion and divergence (Romani et al. 2018). Currently, the number of HD-Zip genes ranges from 15 in *H. annuus* to 180 in *Glycine max* (Table S2). The number of each HD-Zip subfamily also varies widely among plant species. The fewest genes were detected in subfamily I, II, III and IV in *Selaginella moellendorffii* (4), *Spinacia oleracea* (3), *Amborella trichopoda* (7), and *P. patens* (4), respectively (Table S3). In turn, the most genes in subfamilies I, II, III and IV were identified in *Gossypium hirsutum* (57), *Triticum aestivum* (39), *Gossypium hirsutum* (28) and *Solanum lycopersicum* (45), respectively. These differences are due to the polyploidy status of the plant and evolutionary events, including WGDs.

## Phylogeny and Molecular Characteristics of the HD-Zip Subfamily I

**Fig. 2:**
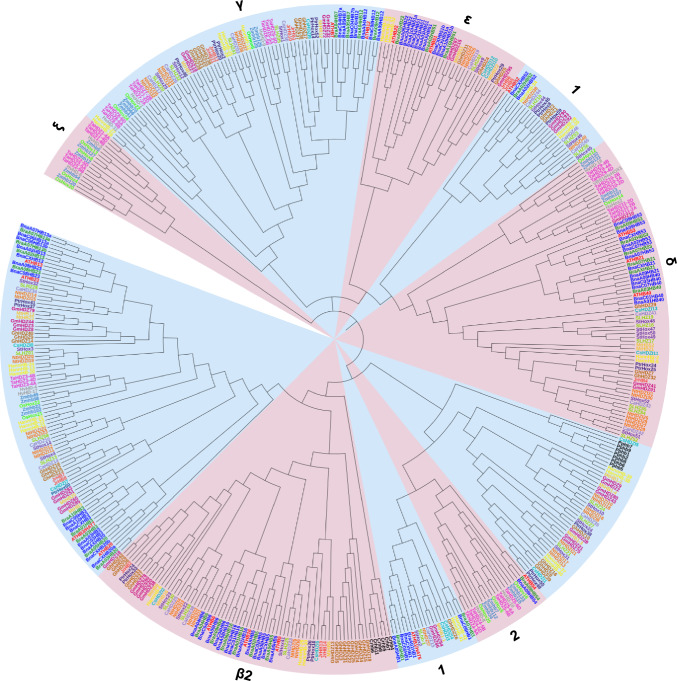
The phylogenetic relationship of the HD-Zip I proteins in plant species based on data collected in PhyloGenes relase (version 3.0) (http://www.phylogenes.org/). Amino acid sequences of the HD-Zip I subfamily were selected from 18 species representing the major plant taxa: *Arabidopsis thaliana*, *Brassica rapa*, *Brassica napus*, *Oryza sativa*, *Hordeum vulgare*, *Solanum lycopersicum*, *Solanum tuberosum*, *Glycine max*, *Nicotiana tabacum*, *Medicago truncatula*, *Triticum aestivum*, *Zea mays*, *Junglas regia*, *Cucumis sativa*, *Helianthus annuus*, *Populus trichocarpa*, *Gossypium hirsutum* and *Capsicum annuum*. The name of genes have been denoted according to the published data: *B. rapa* (Khan et al. 2018)*, O. sativa* (Agalou et al. 2008)*, H. vulgare* (Li et al. 2019a, b)*, S. lycopersicum* (Zhang et al. 2014)*, S. tuberosum* (Li et al. 2019a, b)*, G. max* (Chen et al. 2014)*, N. tabacum* (Li et al. 2019a, b)*, M. truncatula* (Li et al. 2022)*, T. aestivum* (Yue et al. 2018)*, Z. mays* (Mao et al. 2016)*, C. sativa* (Liu et al. 2013)*, P. trichocarpa* (Hu et al. 2012)*, G. hirsutum* (Zhang et al. 2016) and *C. annuum* (Zhang et al. 2021). The phylogenetic tree was constructed using MEGA 11 program with the NJ method and 1000 bootstrap replicates. The analyzed proteins were divided into twelve clades designated α, β1, β2, γ, δ, ε, φ1, φ2, ξ and η, and indicated by a specific color (Henriksson et al. 2005; Li et al. 2019a, b) (Color figure online)

The HD-Zip I subfamily is one of the most abundant groups of homeoboxes, whose members play key roles in plant development and response to environmental conditions (Vlad et al. 2014). Phylogenetic analysis of 483 HD-Zip I proteins of several monocotyledonous and dicotyledonous species confirmed their evolutionary relationships (Fig. 2; Table S4). They are clearly clustered into ten early identified clades: α, β1, β2, γ, δ, ε, φ1, φ2, ζ and η (Henriksson et al. 2005; Li et al. 2019a, b). Similar to previous studies, the clade ζ includes only HD-Zip I proteins from monocotyledons and the clades β2, η and φ2 represent only the HD-Zip I proteins from dicotyledons. The expansion of the HD-Zip I subfamily by WGD was observed in all species studied, including the *Brassica*, *Oryza*, *Gossypium*, *Zea*, *Populus* and *Nicotiana* genera. The HD-Zip I subfamily was the best characterized in the model plant *A. thaliana*. Therefore, in this review, all data on the structure and function of these genes are presented for *A. thaliana* and then related to other species. In *Arabidopsis*, the HD-Zip I subfamily includes 17 members divided into eight clades: α-AtHB3, AtHB20, AtHB13 and AtHB23; β1-AtHB1; β2-AtHB5, AtHB6 and AtHB16; ε-AtHB22 and AtHB51; δ-AtHB21, AtHB40 and AtHB53; γ-AtHB7 and AtHB12; φ1-AtHB52; and φ2-AtHB54 (Fig. S1A). These clades, with the exception of β1, φ1 and φ2, include pairs of paralogous genes located in the WGD-derived duplicated chromosomal regions (Henriksson et al. 2005). Based on the duplication history of the *A. thaliana* genome, the origin of several pairs of paralogous genes *HD-Zip I* has been linked to the major gene duplication events that occurred 20 to 60 million years ago (Blanc et al. 2003). Among them, *AtHB13* and *AtHB23*, *AtHB7* and *AtHB12*, *AtHB6* and *AtHB16*, *AtHB21* and *AtHB40*, and *AtHB3* and *AtHB20* evolved from the recent segmental genome duplication. In contrast, the paralogous genes *AtHB21* and *AtHB53* likely evolved from ancient segmental duplication (Blanc et al. 2003). The phylogenetically related genes show high sequence similarity with the same exon-intron organization and domain topology (Figs. S1B and S1C; Table S5). In particular, the regions corresponding to HD are highly conserved, whereas LZ shows greater sequence variation. Because LZ is a dimerization motif that is a prerequisite for DNA binding, differences within this motif may affect the efficiency of this process. To form dimers required for recognition of the pseudopalindromic sequence CAAT(A/T)ATTG, Zip folds into an α-helical conformation with a leucine residue at every seventh position on the same side of the helix. This structure allows dimer formation through hydrophobic interactions (Landschulz et al. 1988; Chan et al. 1998; Tron et al. 2004). However, other differences in gene structure and functional domains have been observed between distantly related species. As mentioned previously, the *HD-Zip I* genes of Kleobsormidiales show a different exon–intron structure than *Marchantia* and other land plants, including the additional intron within the HD domain (Romani et al. 2018). Also, the fern *Ceratopteris richardii* *HD-Zip I* genes have seven additional amino acids in half of LZ (Aso et al. 1999). In addition, differences were observed in two amino acid residues in LZ between ferns and angiosperms, which are important for dimerization (Sakakibara et al. 2001). This suggests that DNA binding preference and regulatory capability of HD-Zip I members are different in these plant lineage. Recent reports confirmed the important role of additional motifs in the carboxy- and amino- terminal regions (CTR and NTR) that may be involved in protein interaction (Arce et al. 2011; Capella et al. 2014; Fig. S1B). The variability of these motifs may account for the fact that related HD-Zip I proteins, although recognizing and binding to the same pseudopalindromic sequence CAAT(A/T)ATTG, interact with different specific partner proteins to regulate distinct signaling pathways. Among these additional motifs, the AHA motif downstream of LZ form an amphipathic and negatively charged helix contacting components of the basal transcription complex (Döring et al. 2000). Transactivation of CTR was experimentally confirmed for the *Arabidopsis* AtHB1, AtHB7, AtHB12 and AtHB13 proteins (Arce et al. 2011; Capella et al. 2014). Also, in the barley HvHox2 protein, 14 additional amino acids were discovered within CTR that are characteristic for an AHA motif (Sakuma et al. 2010). In contrast, in sunflower HD-Zip I proteins defined the role of the N-terminal flexible arm in DNA–protein interaction (Palena et al. 2001).

## Biological Role of the HD-Zip I Transcription Factors

**Fig. 3:**
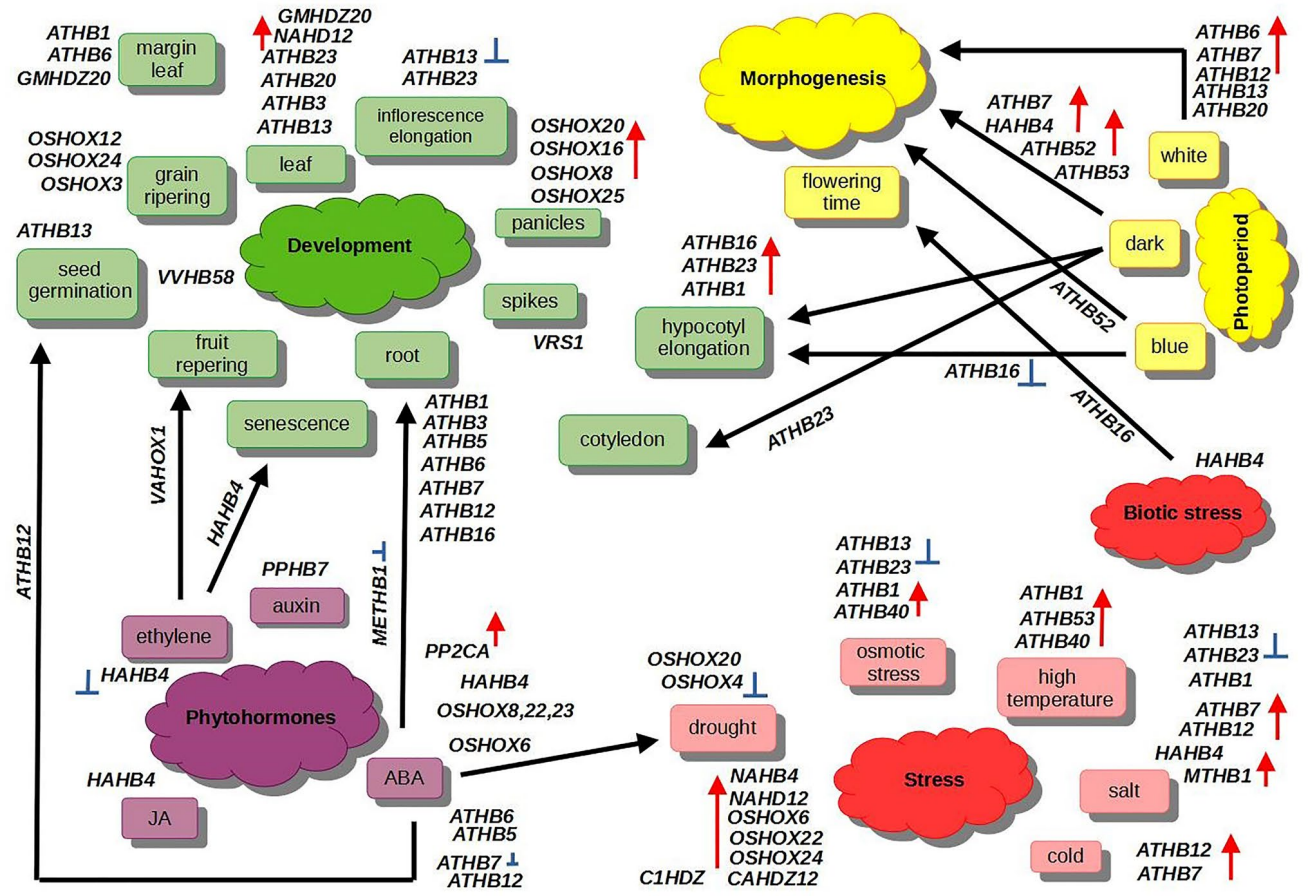
The transcriptional activity of the *HD-Zip I* genes in different biological processes in plants. Positive and negative regulatory actions are indicated by red arrows and blue lines with bars, respectively. Two-letter prefixes for sequence identifiers indicate species of origin: Le-*Lycopersicon esculentum,* At-*Arabidopsis thaliana*, Mt-*Medicago truncatula* (Color figure online)

The first HD-containing gene discovered in plants was the *KNOTTED1* gene of maize, which is involved in leaf differentiation (Vollbrecht et al. 1991). Since then, entire complements of *HD* genes have been identified and functionally characterized in many species, demonstrating their diverse biological functions. The *HD-Zip I* genes are key regulators of plant developmental processes and stress responses (Capella et al. 2015). Transcriptomic studies have shown that they are expressed at different developmental stages and in different organs such as leaves, roots, stems, flowers and fruits, and that they are associated with different external stimuli such as light, phytohormones and stress conditions (Figs. S2 and S3). Some reports confirm that members of *HD-Zip I* can also regulate developmental processes independent of stress conditions (Komatsuda et al. 2007; Gong et al 2019; Sharif et al. 2021). The summary model of the functions of *HD-Zip I* genes is shown in Fig. 3.

### Regulation of Plant Growth and Development Under Different External Stimuli

The *HD-Zip I* genes are key regulators of plant growth and development and control various signaling pathways involved in these processes through interactions with other genes. The mechanisms regulating these processes gradually evolved after the divergence of mosses and vascular plants, i.e. around 430 Mya and contributed to their phenotypic diversity and adaptation to different environmental niches. In the moss *P. patens*, the identified *Pphb7* gene is involved in epidermal cell differentiation and regulates various features of rhizoids under auxin induction (Sakakibara et al. 2003). This function of the *HD-Zip I* gene was not confirmed in flowering plants, so Sakakibara and co-workers proposed that *Pphb7* belongs to a new class of regulators that negatively control total chloroplast mass per cell. In *A. thaliana*, most of 17 *HD-Zip I* genes that arose by WGD events show only partial functional redundancy in plant growth and development (Fig. S2). The comprehensive transcriptional studies confirmed that 4 and 11 members of the *HD-Zip I* subfamily are involved in flower and root development, respectively (Henriksson et al. 2005). *AtHB1*, *AtHB7*, *AtHB12* and *AtHB40* are expressed in flowers. Whereas, *AtHB1*, *AtHB3*, *AtHB5*, *AtHB6*, *AtHB7*, *AtHB12*, *AtHB16*, *AtHB20*, *AtHB21*, *AtHB23* and *AtHB40* are expressed in roots of seedlings growing under well-watered conditions. However, some genes show specific expression patterns and root tissue- or cell-specificity (Perotti et al. 2021, for review). *AtHB3* and *AtHB20*, belonging to the phylogenetic clade α, are up-regulated in roots, and *AtHB5* and *ATHB6* from the phylogenetic clade β2 are down-regulated in roots but up-regulated in the hypocotyl. The paralogous genes *AtHB13* and *AtHB23* are induced in the same root cell types and *AtHB23* is also transcriptionally activated in stem cells and in the early stages of the lateral root primordium (Perotti et al. 2019). Several members of *HD-Zip I *with the highest expression level of *AtHB20* were detected in both root hair and non-hair epidermal cells. In contrast, *AtHB1*, *AtHB5*, *AtHB6*, *AtHB12* and *AtHB16* were expressed in the xylem and *AtHB1*, *AtHB16*, *AtHB51* and *AtHB53* were expressed in the procambium. The paralogous genes *AtHB7* and *AtHB12* also show functional complexity in developmental processes, reflected in different expression patterns and their root tissue specificity. Only *AtHB12* was expressed in lateral root primordia, young leaves and inflorescence stems (Hur et al. 2015; Perotti et al. 2021, for review). These genes are also upregulated in flowers and siliques but expressed at different developmental stages (Olsson et al. 2004; Ré et al. 2014; Ribone et al. 2015; Figs. S2 and S4). Transcriptome analyzes showed that *HD-Zip I* genes in other species are also expressed in different organs and developmental stages, but the evolutionarily related genes show only partial functional similarity (Agalou et al. 2008; Fig. S3). In rice, the *OsHOX6*, *OsHOX8*, *OsHOX22* and *OsHOX23* genes are highly expressed in seedlings. *OsHOX3*, *OsHOX12* and *OsHOX24* are expressed during grain ripening and development, whereas *OsHOX8*, *OsHOX16*, *OsHOX20*, *OsHOX22* and *OsHOX25* are induced during the maturation of panicles (Fig. S3). In cereals, some members of *HD-Zip I* are also involved in developmental processes. For example, the paralogous genes *VRS1* (*HvHox1*) and *HvHox2* of barley have conserved transcriptional activity but show differences in expression, i.e. *VRS1* is expressed in the lateral spikelet primordia in immature spikes, whereas *HvHox2* is broadly induced in different tissues (Komatsuda et al. 2007; Sakuma et al. 2010).

**Fig. 4:**
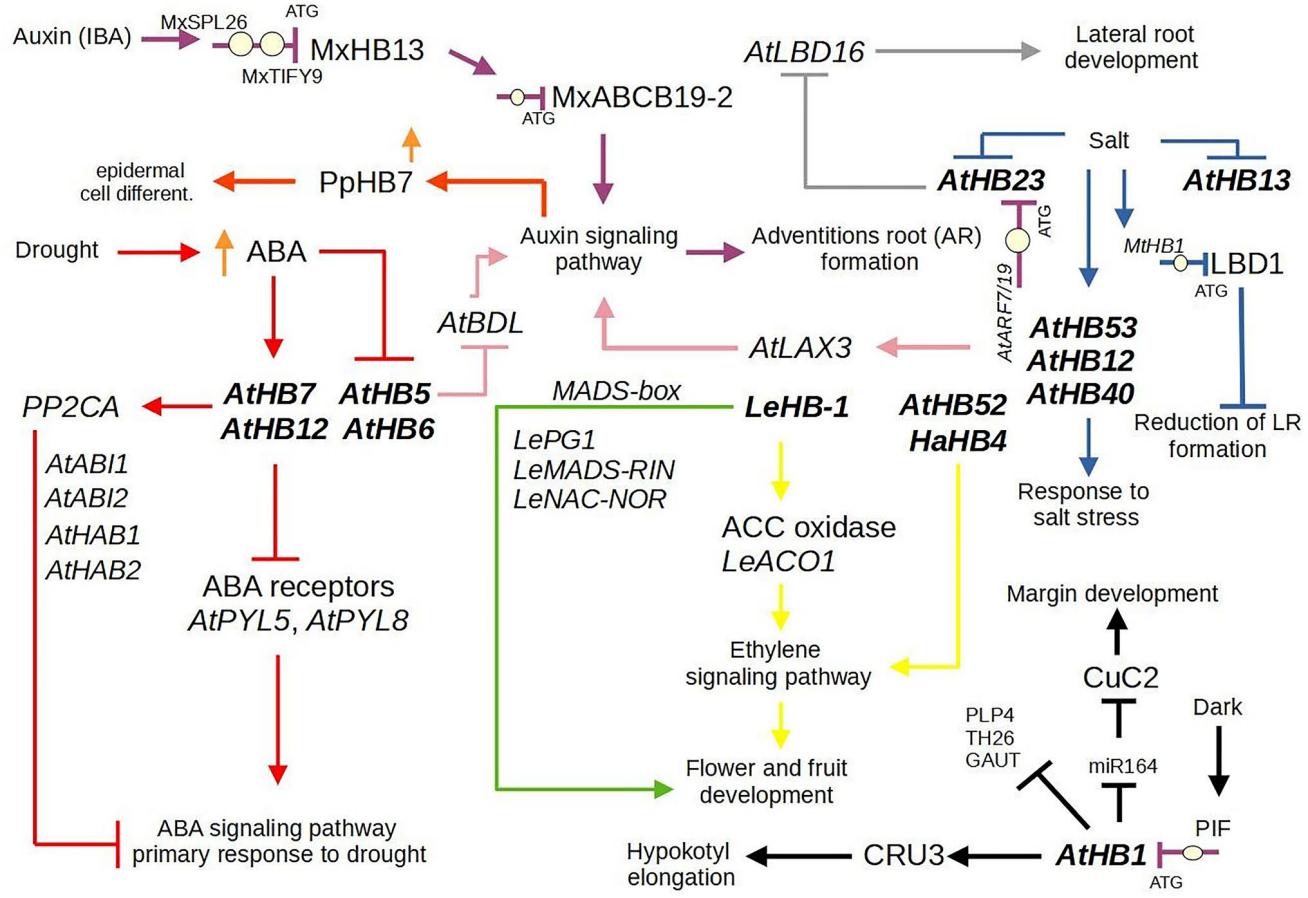
The model of regulation of various biological processes depending on HD-Zip I. Two-letter prefixes for sequence identifiers indicate species of origin: Le-*Lycopersicon esculentum,* At-*Arabidopsis thaliana*, Mt-*Medicago truncatula*

The regulation of plant growth and development is a complex process that requires the integration of multiple factors and signaling pathways and is often controlled by external stimuli. The role of *HD-Zip I* genes in these processes has been investigated using several approaches, including overexpression or RNAi suppression of target genes. The *AtHB1* gene was the first member of this subfamily to be identified in *A. thaliana*, and it proved to be a mediator in determining leaf cell fate in *AtHB1-*overexpressing tobacco lines (Aoyama et al. 1995). The authors found that leaf development controlled by *AtHB1* was dependent on light conditions. They also observed that tobacco plants overexpressing *AtHB1* and growing in the dark exhibited de-etiolated phenotype. However, subsequent studies with *A*. *thaliana 35S::AtHB1* plants and athb1 mutants failed to confirm these results (Capella et al. 2015). The Capella and coworkers suggested that the de-etiolated phenotype observed in tobacco plants may be an artefact due to heterologous expression, controlled by other members of the *HD-Zip I *subfamily or the result of an interaction of *AtHB1* and other *HD-Zip I* genes. In contrast, they showed that *AtHB1* is expressed in the hypocotyl and roots of young seedlings and is involved in hypocotyl elongation under short-day conditions (Capella et al. 2014, 2015; Fig. 3). Based on the above studies, they proposed a model for the regulation of hypocotyl elongation in which PHYTOCHROME-INTERACTING FACTOR 1 (*PIF1*) and *AtHB1* play key roles and which is dependent on light conditions (Fig. 4). According to this model, *PIF1* binds to the promoter of *AtHB1*, which controls downstream expression of other genes to promote hypocotyl elongation under a short-day photoperiod. *AtHB1* induces the expression of *CRU3* and represses the expression of *PLP4* (a patatin-related phospholipase A responsible for cell elongation modifications), *XTH26* (involved in cell wall loosening) and *GAUT* (belongs to the glycosyltransferase family 8 subgroup and is involved in secondary cell wall integrity). Other members of the *HD-Zip I* subfamily are also involved in the regulation of hypocotyl length in response to light conditions. For example, the *AtHB16* and *AtHB23* genes oppositely regulate hypocotyl elongation in response to blue and red light, respectively (Wang et al. 2003; Choi et al. 2014; Fig. 3). The *HD-Zip I* genes are also involved in other developmental processes such as leaf morphology and morphogenesis, root development and regulation of flowering time (Fig. 3). The *A. thaliana AtHB1*, *AtHB6*, *AtHB13* and *AtHB16* genes are involved in the regulation of leaf shape, including the promotion of leaf margins, but they control their different components (Wang et al. 2003; Lechner et al. 2011; Gao et al. 2014; Miguel et al. 2020). For example, *AtHB1* regulates *CUC2* expression by specifically repressing miR164 levels to promote leaf margins (Miguel et al. 2020; Fig. 4). The crucial role in regulating leaf development was also confirmed for *AtHB3*, *AtHB13*, *AtHB20* and *AtHB2*3 in transgenic *A. thaliana* overexpressing these genes (Henriksson et al. 2005). The paralogous genes *AtHB13* and *AtHB23* have partially similar functions in inhibiting inflorescence stem elongation but show no functional redundancy in other biological processes (Ribone et al. 2015). *AtHB23* is expressed in the adaxial region of leaves and is involved in hypocotyl elongation and cotyledon expansion under red light (Kim et al. 2007; Choi et al. 2014). Whereas, *AtHB13* is involved in stress responses, pollen hydration and seed germination (Cabello and Chan 2012; Gao et al. 2014; Ribone et al. 2015).

Many orthologs of *A. thaliana AtHB* genes identified in different plant species have been shown to be involved in the regulation of plant growth and development (Figs. 2 and S3). However, despite their evolutionary relationship, the orthologs exhibit only partial functional similarity at the transcriptional level. Many of them have evolved novel tissue preferences that have led to changes in morphological traits and facilitated adaptation to different environmental conditions. The *Medicago truncatula MtHB1* gene, which is an ortholog of *A. thaliana AtHB7* and *AtHB12*, is also induced in roots under exogenous ABA treatment and salt stress, resulting in inhibition of lateral root growth (Ariel et al. 2010). *AtHB13* and its orthologous gene *HaHB1* in sunflower are involved in leaf development. The transgenic plants overexpressing these genes show similar morphological characteristics such as serrated leaves and a differentiated cotyledon phenotype under sucrose 4% (Hanson et al. 2002). In contrast, orthologs of *AtHB1* gene showed only partially conserved functions in organ development. The tomato *LeHB-1* gene is involved in the flowering process by regulating floral organogenesis, carpel development and ripening (Lin et al. 2008). The grape *VvHB58* gene controls fruit size, seed number and inhibits pericarp expansion in tomato by modulating various hormonal pathways (Li et al. 2019a, b). Whereas, the soybean *GmHDZ20* gene is involved in regulating leaf morphology and the number of seeds per pod (Yang et al. 2020). Interestingly, the complex role of the *Mict* gene of *Cucumis sativus*, which belongs to the *HD-Zip I* subfamily, was found in multicellular trichome development (Zhao et al. 2015). At an early stage of trichome development, *Mict* negatively and positively controls trichome spacing and base cell morphogenesis, respectively. At later stages, *Mict* positively controls apical cell and stalk cell morphogenesis. A spectacular example of functional diversity becoming visible as phenotypic variation was observed in the paralogous genes *VRS1* (*HvHox1*) and *HvHox2* in barley (Sakuma et al. 2010). Phylogenetic analysis showed that *VRS1* is a paralogue of *HvHox2* that arose by duplication of an ancestral gene (Sakuma et al. 2010; Fig. 2). However, during the evolution of barley (after the separation of the taxa *Brachypodium* and Pooideae), *VRS1* acquired a new function restricted to the immature inflorescence. By comparing the sequences of both genes, a mutation in the third exon of *VRS1* was found to contain a conserved motif at the C-terminus of HvHox2, resulting in its new functions (Sakuma et al. 2010). More detailed studies showed that loss of function of the *VRS1* gene led to a complete conversion of the rudimentary lateral spikelets from a two-row to a six-row phenotype (Sakuma et al. 2010, 2013).

### Hormone Signal Transduction and Stress Responses

As sessile organisms, plants had to develop new mechanisms to adapt to the rapidly changing environment. Plant stress responses controlled by transcription factors such as HD-Zip I are often associated with developmental plasticity resulting from the interplay of morphology and growth rate and hormone signaling pathways. The *HD-Zip I* genes show expression changes mainly in response to treatment with abscisic acid (ABA), known stress hormone in plants (Henriksson et al. 2005). In *A. thaliana*, most members of this subfamily are induced or repressed in response to exogenous ABA and various stresses such as drought, heat, salinity and cold (Fig. S4). However, due to their functional diversity, they show different responses to these stimuli. Some reports have highlighted the complex interactions between the HD-Zip I transcription factors and stress, which can be attributed to evolutionary events (Lee et al. 2001; Himmelbach et al. 2002; Olsson et al. 2004). There is a correlation between the function and evolutionary relatedness of the members of this subfamily. The paralogous gene pair *AtHB40* and *AtHB53* is induced by high temperature, osmotic stress and salinity (Perotti et al. 2021; Fig. 3). The paralogous gene pairs *AtHB7* and *AtHB12* and *AtHB5* and *AtHB6* are regulated by water deficit conditions in an ABA-dependent pathway (Söderman et al. 1999; Lee et al. 2001; Himmelbach et al. 2002; Olsson et al. 2004; Fig. 3). The *AtHB7* and *AtHB12* genes are induced by exogenous treatment with ABA, but their expression patterns differ depending on the developmental stage of the plant (Olsson et al. 2004). Only *AtHB12* shows sensitivity of ABA during seed germination and reduces inflorescence stem growth by inhibiting hormone synthesis of gibberellic acid (GA) (Son et al. 2010). Further studies showed that they act as negative regulators in the ABA-dependent response to drought (Johannesson et al. 2003; Deng et al. 2006; Valdes et al. 2012). Thus, *AtHB7* and *AtHB12* are regulators of plant growth and development controlled by ABA and drought stress. The *Arabidopsis* *HD-Zip I* genes are also expressed under other stress conditions and show opposite expression patterns. The paralogous genes *AtHB7* and *AtHB12* are up-regulated under cold and salinity, whereas the paralogous genes *AtHB13* and *AtHB23* are repressed under osmotic stress and salinity (Perotti et al. 2021; Fig. 3). The authors also concluded that *AtHB23* is essential for plant survival and adaptation to salt stress conditions by controlling the gravitropic response mediated by starch granule turnover. These results confirm that duplication followed by structural rearrangements of the *HD-Zip I* genes is responsible for their partial functional diversity toward opposing activities in repressing the ABA signaling network. Other members of* HD-Zip I* are also up- or down-regulated depending on stress conditions. *AtHB1*, for example, is induced in response to osmotic stress, UV-B, and high temperature, but is suppressed upon NaCl treatment (Perotti et al. 2021).

The biological functions of *HD-Zip I* genes associated with the stress response have also been studied in other monocotyledonous and dicotyledonous plants. Expression profiling of these genes revealed that many of them are induced by ABA, drought, cold and salt stresses. Despite their evolutionary relationship with the *Arabidopsis* *HD-Zip I* genes, they show only partially similar expression patterns under these stress conditions (Fig. S5). The orthologous genes of *AtHB7* and *AtHB12* have been identified and functionally characterized under abiotic stress conditions in other species such as sunflower, rice, tobacco and chickpea (Gago et al. 2002; Agalou et al. 2008; Ré et al. 2011; Sen et al. 2017). The studies based on the overexpression and repression of these genes show that only some of them retained their ancestral functions in regulating stress responses. The *HaHB4* gene of *H*. *annuus* is involved in hormone-dependent responses (Gago et al. 2002). Similar to the *AtHB7* and *AtHB12* genes of *A. thaliana*, it is up-regulated in response to ABA and drought (Lee and Chun 1998). However, later studies show that only the *HAHB4* gene confers drought tolerance to transgenic *Arabidopsis* plants under the control of constitutive or drought-inducible promoters (Dezar et al. 2005). This is related to its ability to protect plants from photooxidative stress by down-regulating the biogenesis of photosynthetic machinery (Manavella et al. 2006). Its expression also changes in response to jasmonic acid, mechanical damage, mannitol and ethylene during *Spodoptera* sp. attack (Dezar et al. 2005; Manavella et al. 2006, 2008). Detailed analyzes revealed that *HAHB4* is an integral component of the phytohormone signaling pathways JA, ET and SA that regulate the response to biotic stress. Overexpression of *HaHB4* also resulted in the decreased expression of ethylene signaling and biosynthesis genes confirming its involvement in the regulation of flower and fruit development (Manavella et al. 2006; Fig. 3). The *Nicotiana attenuata NaHD12* gene is highly expressed in roots and leaves under exogenous ABA treatment and drought stress (Ré et al. 2011). The *nahd20* mutant showed decreased expression of the *NaNCED1* (ABA biosynthesis gene) and *NaOSM1* (osmotin 1) genes under water deficit. In rice, the orthologous *OsHOX6*, *OsHOX22* and *OsHOX24* genes of the *AtHB7* and *AtHB12* genes of *A. thaliana* are also induced by drought stress, but their expression differs from that of their counterparts in *Arabidopsis* and also from each other (Agalou et al. 2008). The *OxHOX6* gene shows high expression in all tissues under well-watered conditions and is upregulated under drought in the drought-sensitive rice cultivars. Whereas, the more closely related *OxHOX22* and *OsHOX24* genes are strongly expressed in leaves and panicles and only weakly expressed in other tissues under well-watered conditions (Fig. S3). Overexpression of these genes in the *A. thaliana* background resulted in the susceptibility of these plants to ABA, drought and salt at different developmental stages, confirming their negative regulation of response to these stressors (Agalou et al. 2008; Bhattacharjee et al. 2016). The negative role of *HD-Zip I* genes in stress responses has also been confirmed in other species. Overexpression of *Jatropha curcas JcHDZ07* in transgenic *A. thaliana* plants increases their sensitivity to salt stress (Tang et al. 2019). RNAi suppression of the tomato *SlHB2* gene also confirmed its role as a negative regulator of salt and drought stress responses (Hu et al. 2017). On the other hand, the positive role of *HD-Zip I* genes in regulation of salt stress response was also confirmed, but they are controlled by other mechanisms. Overexpression of the maize *ZmHDZ10* gene in both *A. thaliana* and rice leads to increased tolerance to drought and salinity in these plants by ABA-dependent signal transduction (Zhao et al. 2014). Similarly, overexpression of the *MtHB1* gene in *M. truncatula* plants had a positive effect on increasing their salinity tolerance through the root system control (Ariel et al. 2010). The main regulatory mechanism of this process is associated with the repression of the LOB BINDING DOMAIN (*LBD1*) gene by *MtHB1* under salt stress and ABA treatment during root formation (Fig. 4). In apple, overexpression and RNAi suppression of the *Malus domestica MdHB7*-like gene showed that it is involved in the regulation of salt tolerance by controlling sugar accumulation (Zhao et al. 2021). In contrast, overexpression of chickpea *CaHDZ12* in tobacco increased tolerance to osmotic stress by reducing the accumulation of ROS and sensitivity to ABA (Sen et al. 2017). The complex functions of the *HD-Zip I* transcription factors are also observed in the “resurrection plant” *Craterostigma plantagineum*. The *CpHB4* and *CpHB5* genes grouped in the phylogenetic tree with *AtHB5*, *AtHB6*, and *AtHB16* and *AtHB1*, respectively, show down-regulation in leaves and roots during drought stress (Deng et al. 2002; Harris et al. 2011). Interestingly, the *CpHB6* and *CpHB7* genes grouped with *AtHB52* and *AtHB54* show different expression patterns (Deng et al. 2002; Harris et al. 2011). They are upregulated under drought stress, and the expression of *CpHB7* is similar to that of *AtHB6* under normal growth conditions (Deng et al. 2006). These results indicate that the function of some members of HD-Zip I is partially conserved in *Arabidopsis* and *Craterostigma*. In contrast, no correlation between phylogenetic relationship and functional conservation of *HD-Zip I* genes was found in *Marchantia polymorpha*, an early-divergent bryophyte, and *A. thaliana*. The *MpC1HDZ* gene is the single-copy ortholog with all the features of the *HD-Zip I* genes found in *A. thaliana*. This gene plays a specific role in biotic stress response not found in its orthologs in flowering plants (Romani et al. 2020). Romani and coworkers found that the functions of this gene are related to oil body formation, terpenoid biosynthesis and response to herbivory, but not to the regulation of response to abiotic stress. These studies clearly show that phylogenetic relationship of genes based on sequence similarity does not correlate with their functional conservation in evolutionarily distant species such as early-diverging land plants and angiosperms. One of the reasons for this functional diversity could be the WGD events that were followed by diploidization and genome rearrangements in flowering plant lineage, where newly formed genes acquired new functions.

### Photomorphogenesis

Changes in plant growth and development occur frequently under light stimuli and are controlled by both the amount and wavelength of light. They are caused by information received from photoreceptors and the cascade of light-induced (or light-independent) responses leads to changes in gene transcription. Some members of *HD-Zip I* are involved in the modulation of light-dependent signaling networks (Henriksson et al. 2005; Fig. 3). The authors found that the expression of the *AtHB52* gene is regulated by light, therefore it may be involved in photomorphogenesis (Fig. 3). Its expression was increased 30-fold in darkness compared to white light and four-fold in blue light. In contrast, the expression of *AtHB53* is upregulated in darkness but not under blue light conditions. Transcript levels of *AtHB5, AtHB6, AtHB7, AtHB12, AtHB13* and *AtHB20* are higher in seedlings grown under white light than under blue light or in darkness. In other work, *AtHB16* was found to be a component of light sensitization mechanisms in plants (Wang et al. 2003). This study confirmed the role of *AtHB16* as a suppressor of flowering time sensitivity to photoperiod. The transgenic *Arabidopsis* plants with reduced levels of *AtHB16* showed enhanced response of flowering time to photoperiod (Fig. 3). Moreover, this transcription factor may control plant development as a mediator of a blue light response by positively regulating blue light-dependent inhibition of hypocotyl growth. Other HD-Zip I, AtHB23 interacts with phytochrome B (phyB) and is involved in phyB-mediated light signal transduction in *Arabidopsis* (Choi et al. 2014). Molecular analysis of the *Arabidopsis athb23* mutant revealed altered hypocotyl growth under R light and defects in phyB-dependent seed germination and cotyledon expansion. Choi and coworkers (2014) hypothesized that *ATHB23* is a novel component of the phyB-mediated R-light signaling pathway.

Recent reports have shown that interactions between different HD-Zip families link both morphogenesis and environmental responses through phytohormone signaling pathways. Brandt and coworkers (2014) found that members of the *HD-Zip I *subfamily *AtHB7* and *AtHB12* and *REV*, which belong to *HD-Zip III*, oppositely regulate ABA-receptor proteins in a cell type specific manner.

## Molecular Mechanisms of Target Gene Regulation by HD-Zip I Transcription Factors

The main role of TFs is to regulate cell- and tissue-specific gene expression by binding to the specific sequence in the promoters of target genes or by physically interacting with other proteins. The major functional elements of the HD-Zip I proteins are the well-characterized HD and LZ, which are responsible for DNA binding and dimerization, respectively. The *HD-Zip I* transcription factor specifically recognizes and binds the pseudopalindromic sequence CAAT(A/T)ATTG in promoters of target genes (Palena et al. 2001; Table S6). Recently, using the DAP-seq technique for in vitro TF-DNA binding assays, the pseudopalindromic sequence AAT(N)ATT was identified as a target for* HD-Zip I* (O’Malley et al. 2016). The DNA-binding process requires dimerization by Zip. Many of the HD-Zip I proteins are capable of forming homodimers, but some of them such as AtHB5, AtHB6, AtHB7, AtHB12 and AtHB16 also have the ability to form heterodimers with different affinities, depending on structural constraints (Johannesson et al. 2001). DNA-binding site preference for HD-Zip I is determined by residues in the helix III and the N-terminal arm of HD (Palena et al. 2001). Interestingly, the different binding preferences of the closely related AtHB7 and AtHB12 for this consensus motif have been demonstrated *in planta* and in vitro (Johannesson et al. 2001; Henriksson et al. 2005; Valdes et al. 2012). *AtHB7* and *AtHB12* do not interact in vitro with the CAATNATTG sequence recognized by *AtHB1*, *AtHB3*, *AtHB5*, *AtHB6*, *AtHB13* and *AtHB16*, despite high sequence similarity in the DNA binding assay (Johannesson et al. 2001). On the other hand, they bind to this sequence *in planta* and activate transcription (Henriksson et al. 2005; Valdes et al. 2012). Johannesson and coworkers suggest that the differences in phosphorylation sites in HD between AtHB7 and AtHB12 and other HD-ZIP I proteins may involve posttranslational modifications and affect DNA binding preference. Although most HD-Zip I proteins bind to the same DNA sequence, they exhibit different expression patterns and regulatory properties. Therefore, other functional elements are also considered that may be involved in the interaction with target proteins and affect the different functions of these proteins. These include additional motifs in the CTR and NTR regions of the HD-Zip I proteins that could play an activating or regulatory role by physical interaction with different specific partner proteins (Fig. S1B).

Recently, a general model for the organization of functional regions in the HD-Zip I proteins was proposed (Arce et al. 2011). According to this model, they characterize the presence of HD, Zip, AHA motif in the C-terminal region and regulatory motifs in the N- and C-terminal regions. Although HD and LZ are highly conserved, the N- and C-terminal regions show differences and not all motifs were found in each protein (Fig. S1B). The variability of the N- and C-terminal regions could possibly lead to the differences in the activity of the members of HD-Zip I. The number and distribution of regulatory motifs correlate with phylogenetically distinct groups within the HD-Zip I subfamily (Fig. 2). The importance of HD and LZ for protein-protein interaction has been experimentally confirmed for AtHB6 and AtABI1 (Abscisic acid Insensitive 1) and AtHB6 and BPM3, which belongs to the MATH-BTB family, among others (Himmelbach et al. 2002; Lechner et al. 2011). The functional domain of AtABI1 and a serine residue within the HD of AtHB6 were found to be responsible for their physical interaction. The LZ of AtHB6 is recognized by BPM3 and this complex is identified by Cullin-RING E3 ubiquitin ligases 3 (CRL3), which facilitates the transfer of ubiquitin moieties to AtHB6 and initiates its degradation by the 26S proteasome. A similar direct physical interaction was confirmed in vivo between AtHB12 and AtABI2 (Valdes et al. 2012). These results suggest that post-translational modifications and protein–protein interactions influence the functions of HD-Zip I. Regulatory motifs located in the N- and C-terminal regions also play important role in these processes. For example, the different versions of the AHA motif were also found in the C-terminal region of the *Arabidopsis* proteins AtHB1, AtHB7, AtHB12 and AtHB13, which act as transcriptional activators (Lee et al. 2001; Fig. S1B). Moreover, this motif may be involved in the transactivation process, as exemplified by AtHB1 by using a yeast one-hybrid approach (Arce et al. 2011). Previous studies have shown that the AHA motif is involved in interactions with proteins representing the basal transcription machinery (Döring et al. 2000; Kotak et al. 2004). The later experimental studies showed that AtHB1 interacts with AtTBP2, AtHB12 with AtTFIIB and AtHB7 with AtTBP2 and AtTFIIB (Capella et al. 2014). In barley, amino acids characteristic of the AHA motif were detected in the C-terminal region of the HvHox2 protein (Sakuma et al. 2010). Interestingly, this sequence has been lost in the closely related VRS1 protein, contributing to a *tl* mutation mimicking effect (Hofer et al. 2009). Therefore, motifs such as AHA could be a potential source of functional divergence between members of the HD-Zip I TFs. Many acidic serine-rich motifs have also been found in the C- and-N-terminal regions of HD-Zip I proteins, which may be involved in activation and phosphorylation or sumoylation processes (Arce et al. 2011). The proximal region of the C-terminal region (adjacent to the LZ) is rich in serine residues that are putative phosphorylation sites (Arce et al. 2011). In addition, putative sumoylation sites were found (Arce et al. 2011). Some potential residues for these posttranslational modifications were also found in the N-terminal region of these proteins. The functions of the C-terminal region in activation and phosphorylation processes for HD-Zip I proteins have been studied experimentally. For example, the phosphorylation of AtHB6 by PKA kinase inhibits its DNA-binding activity in vitro (Himmelbach et al. 2002). These studies suggest that posttranslational modifications such as protein phosphorylation and sumoylation may be important in controlling the activities of HD-Zip I proteins.

The binding of *HD-Zip I* to the promoters of target genes has been studied experimentally, but little is known about the downstream target genes regulated by these transcription factors. However, protein-DNA binding is dependent on external conditions. For example, the binding of HD-Zip I proteins to the upstream regulatory sequences of key components of the ABA signaling pathway, such as PP2CA, ABA receptors and SNF1-related Ser/Thr kinases (SnRKs) is ABA-dependent (Valdes et al. 2012). Valdes and coworkers showed that the binding of AtHB7 and AtHB12 proteins to the promoters of *AtABI1* and *AtABI2* is restricted to plants treated with ABA (Fig. 4). Moreover, the AtHB12 protein binds to the upstream regulatory sequences of *AtHAB1, AtHAB2, AtAHG3 AtPYL5*, *AtPYL8*, *AtSnRK2.3* and *SnRK2.8* only under the same stimulus conditions. In contrast, binding of the AtHB7 protein to the promoters of these genes is ABA-independent. In other studies, the binding of tomato *LeHB-1* to the promoter of *LeACO1* (ACC oxidase), a component of the ethylene biosynthesis pathway, was detected by a gel retardation assay (Lin et al. 2008). Further experimental analyses showed that *LeHB-1* positively controls *LeACO1* and that this interaction is important for the regulation of the ripening process (Fig. 3). Bioinformatic analyzes indicated putative *LeHB-1* binding sites in the promoters of other ripening related genes such as *LePG1*, *LeMADS-RIN*, and *LeNAC-NOR*. This suggests that *LeHB-1* may be involved in the control of flower development by other regulatory factors such as MADS-box genes (Fig. 3). In *Medicago*, *MtHB1* represses LOB-binding domain 1 (*LBD1*), which forms the lateral root emergence phenotype (Ariel et al. 2010, Fig. 4). *LBD1* is involved in auxin-regulated initiation of lateral roots and root formation in *Arabidopsis* and rice, respectively (Liu et al. 2005; Okushima et al. 2007). Recently, *AtHB23* was also found to be involved in the gene regulatory network controlling root branching (Perotti et al. 2019). Perotti and coworkers (2019) observed by chromatin immunoprecipitation-quantitative polymerase chain reaction (ChIP-qPCR) that *AtHB23* directly controls the functions of Lateral Organ Boundary (*LBD16*) and the auxin transporter gene *LAX3* (Fig. 4). It suppresses the expression of *LBD16*, which is a critical element of lateral root development. Perotti and coworkers (2019) observed that *AtHB23* is expressed in the early stages of secondary primordia and later represses *LBD16* in the tertiary primordium lateral root development, further inhibiting root formation. *AtHB23* also induces the auxin transporter gene *LAX3*. The researchers hypothesized that *AtHB23* mediates the regulation of *LAX3* by *ARF7*/*19* (auxin response factors) (Fig. 4). Previously, *AtHB5* was known to be a negative regulator of *BDL* (BODENLOS) expression and to control the *BDL*-dependent auxin response (De Smet et al. 2013; Fig. 4). These data clearly demonstrate that at least some HD-Zip I transcription factors are involved in regulating developmental processes via the auxin signaling pathway.

The HD-Zip I transcription factors can act as positive or negative regulators, which was confirmed in an in vivo promoter-reporter gene assay (Henriksson et al. 2005; Harris et al. 2011; Valdes et al. 2012). For example, *AtHB5* induces transcription, and its close relative *AtHB6* and the other paralogous gene pair *AtHB7* and *AtHB12* are both transcriptional activators and repressors (Himmelbach et al. 2002; Valdes et al. 2012). The authors reported the transcriptional relationships between group A protein phosphatase 2C (*PP2CA*) and *HD-Zip I* genes in drought- and ABA-mediated responses. These studies showed that *AtHB6*, *AtHB7*, and *AtHB12* regulate the ABA signaling pathway in the primary drought response. They positively regulate the expression of *PP2CA* genes, including *ABI1*, and are required for their full transcriptional activity. In addition, *AtHB7* and *AtHB12* repress transcription of the ABA receptor genes *PYL5* and *PYL8*, becoming a negative regulator of the ABA signaling pathway (Fig. 3). Taken together, these data provided evidence for the role of *HD-Zip I* genes in negatively regulating the ABA signaling pathway by upregulating *PP2CA* genes and downregulating ABA-receptor genes. Of note, other members of the HD-Zip I subfamily are also involved in the regulation of ABA signaling. However, this process might be more complex because their expression patterns differ depending on the stage of plant development and external conditions. For example, *AtHB5* and *AtHB20* are also negative regulators of ABA signal transduction during germination (Johannesson et al. 2003; Barrero et al. 2010). These overlapping functions of these genes in regulating ABA signal perception should be further investigated.

## Conclusions and Future Perspectives

The HD proteins act as transcription factors and activate or repress the expression of targets in all living organisms. Their characteristic structural feature is the highly conservative HD domain of 60 amino acids. Together with access to numerous genome sequences, we have explored their evolutionary fate over hundreds of millions of years. Of the 11 families in the HD superfamily, only the HD-Zip family is unique in plants. The major regulatory elements of the HD-Zip proteins are the HD domain and the adjacent LZ motif, which are involved in regulating gene expression by binding to targets in gene promoters and interacting with other proteins, respectively. Recent reports suggest the presence of additional domains, motifs, or cofactors that may be required to increase the specificity of their functions. This suggests that there is not only one mechanism and one region in the HD-Zip proteins that regulate the functions of target genes. In plants, whole genome duplications (WGDs) and additional mechanisms also play important roles in the functional renewal of these transcription factors, which in turn may be responsible for drastic changes in an entire cascade of downstream target genes. Most HD-Zip proteins interact with multiple partners that are the same or different members of this subfamily (homo- or hetero-interactions) or with other regulatory factors such as other transcription factors, signaling molecules, or regulatory RNAs. In addition, the functional divergence of paralogs forms a complex network of interactions, for example, during stress. Therefore, the regulation of gene function is still poorly understood. Further studies should provide new insights into the complexity of gene regulation resulting from the functional differentiation of transcription factors such as homeobox genes.

### Supplementary Information

Below is the link to the electronic supplementary material.

Supplementary Fig. S1. The phylogenetic and structural characterization of the HD-Zip I subfamily in *A. thaliana*. A. The phylogenetic tree of *Arabidopsis* HD-Zip I subfamily based on alignment of full-length protein sequences by using MAGA 11, B. Structural domain organizations of *Arabidopsis* HD-Zip I proteins including the conserved domain (HD and Zip) and additional motifs. Uncharacterized motifs outside the HD and Zip as a potential source of functional diversity are numbered according to Arce et al. (2011). C. Schematic diagram representing the structure of *Arabidopsis HD-Zip I* genes. Yellow boxes represent exons and spaces between boxes correspond to introns. The 5’ and 3’ UTRs are marked by blue boxes. The exon/intron structure of each *HD-Zip I* gene was defined by comparison of their genomic and cDNA sequences. The sizes of exons, introns and UTRs are drawn to scale as indicated below. The gene structures were illustrated using the Gene Structure Display Server (http://gsds.cbi.pku.edu.cn/ (DOCX 295 KB)

Supplementary Fig. S2. Expression profiles of 17 *HD-Zip I* genes in different tissues and stages of development of *A. thaliana*. The data are based on Arabidopsis eFP Browser (The BAR and other Data Analysis Tools for Plant Biology (utoronto.ca). The heatmap shows the log2-transformed TPM values of each gene. The expression level of *AtHB* genes is represented using color scale ranging from green (low expression) to red (high expression (JPG 109 kb)

Supplementary file3 Fig. S3. Expression profiles of *HD-Zip I* genes in five tissues of the selected plant species. The tissues include leaves, stem, roots, flowers and silique/seeds. The Reads/Kb/Million (RPKM) normalized values of expressed genes were log2-transformed and visualized as heatmap. The order of genes in the heatmap is consistency with the phylogeny in Figure 2. The data were from the different databases or the published studies. Data were obtained from: *H. vulgare* - Barley RNA-seq Database morexGenes - Barley Assembly and RNA-seq (hutton.ac.uk); *G. max* - soybean expression atlas (Soybean expression atlas | Home (uenf.br)); *S. lycopersicum* - Tomato BARePlant (ePlant (utoronto.ca)); *L. tuberosum* - the PGSC database (Spud DB (uga.edu)); *Z. mays* - Maize eFP Browser (Maize eFP Browser (utoronto.ca)); *M. truncatula* - Medicago eFP Browser (Medicago eFP Browser (utoronto.ca)); *B. napus* - BrassicaEDB - A Gene Expression Database for Brassica Crops (BrassicaEDB - A Gene Expression Database for Brassica Crops (biodb.org)); *B. rapa* - the Brassicaceae Database (BRAD)(BRAD (brassicadb.cn)); *O. sativa* - Rice eFP Browser (https://bar.utoronto.ca/efprice/cgi-bin/efpWeb.cgi) (PPTX 968 KB)

Supplementary *file4* Fig. S4. Expression profiles of *HD-Zip I* genes under abiotic stresses and ABA treatment of *A. thaliana*. The data are based on Arabidopsis eFP Browser (The BAR and other Data Analysis Tools for Plant Biology (utoronto.ca). The expression level are based on the log2 values and color scale represent the expression levels of *AtHB* genes from green (downregulated) to red (upregulated) (JPG 99 kb)

Supplementary *file5* Fig S5. Expression profiles of *HD-Zip I* genes in response to cold, heat, drought and salt stresses in different species. The Reads/Kb/Million (RPKM) normalized values of expressed genes was log2-transformed and visualized as heatmap. The order of genes in the heatmap is consistency with the phylogeny in Figure 2. Data were obtained from: *B. napus* - BrassicaEDB - A Gene Expression Database for Brassica Crops (BrassicaEDB - A Gene Expression Database for Brassica Crops (biodb.org)); *B. rapa* - based on data from Khan et al. (2018); *G. max* obtained from the NCBI GEO database under accession numbers GSE40627 (drought), GSE41125 (salinity) and GSE213479 (heat); *H. vulgare*: obtained from the NCBI GEO database under accession numbers PRJEB18276 (cold), PRJNA324116 (heat), PRJNA439267 (drought) and PRJEB13621 (salt); *S. lycopersicum* - Tomato BARePlant (ePlant (utoronto.ca)) and obtained from the NCBI GEO database under accession number PRJNA730730 (heat); *L. tuberosum* - the PGSC database (Spud DB (uga.edu)); *Z. mays* - Maize eFP Browser (Maize eFP Browser (utoronto.ca)); *M. truncatula* - based on data from Li et al. (2022); *P. trichocarpa* - Poplar eFP Browser https://bar.utoronto.ca/eplant_poplar/; *C. sativa -* Cucurbit Genomics Database: Cucurbit Genomics Database (CuGenDB); *O. sativa* - Rice eFP Browser (https://bar.utoronto.ca/efprice/cgi-bin/efpWeb.cgi) (PPTX 186 KB)

Supplementary *file6* Table S1. Number of transcription factors (TFs) in plant genomes based on data derived from PlantTFDB v5.0 (http://planttfdb.gao-lab.org/) and PLAZA project (https://bioinformatics.psb.ugent.be/plaza/) (DOCX 35 KB)

Supplementary Table S2. Numbers of *HD-Zip* genes in plant genomes (DOCX 16 KB)

Supplementary *file8* Table S3. The number of genes within each group of the *HD-Zip* family in plants (DOCX 15 KB)

Supplementary Table S4. List of HD-Zip I proteins included in the phylogenetic analysis (XLSX 25 KB)

Supplementary Table S5. Characterization of 17 *A. thaliana* *HD-Zip I* genes including gene and protein lengths, gene and protein organizations and biological functions (DOCX 16 KB)

Supplementary Table S6. Characterization of 17 members of *A. thaliana* HD-Zip I subfamily including the consensus sequence of *cis*-regulatory element and their interacting partners. The sequence logo of cis-regulatory elements were generated by JASPAR (https://jaspar.genereg.net/) (DOCX 76 KB)
