# Supplementary figures and images for: Evolutionary Consequences of Functional and Regulatory Divergence of HD-Zip I Transcription Factors as a Source of Diversity in Protein Interaction Networks in Plants
Source: J Mol Evol. 2023 Jun 23;91(5):581–97. doi: 10.1007/s00239-023-10121-4 (PMC10598176; doi:10.1007/s00239-023-10121-4)

Figure S1

1.
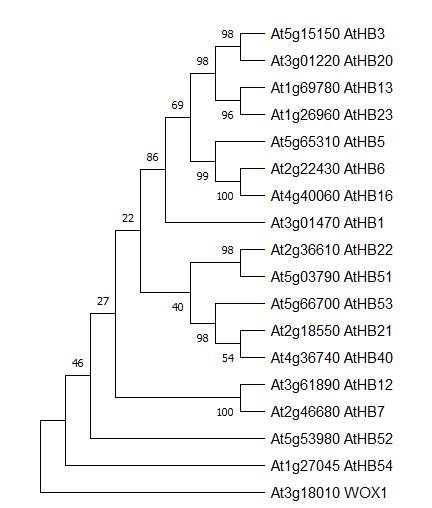
 B.

**Clade**


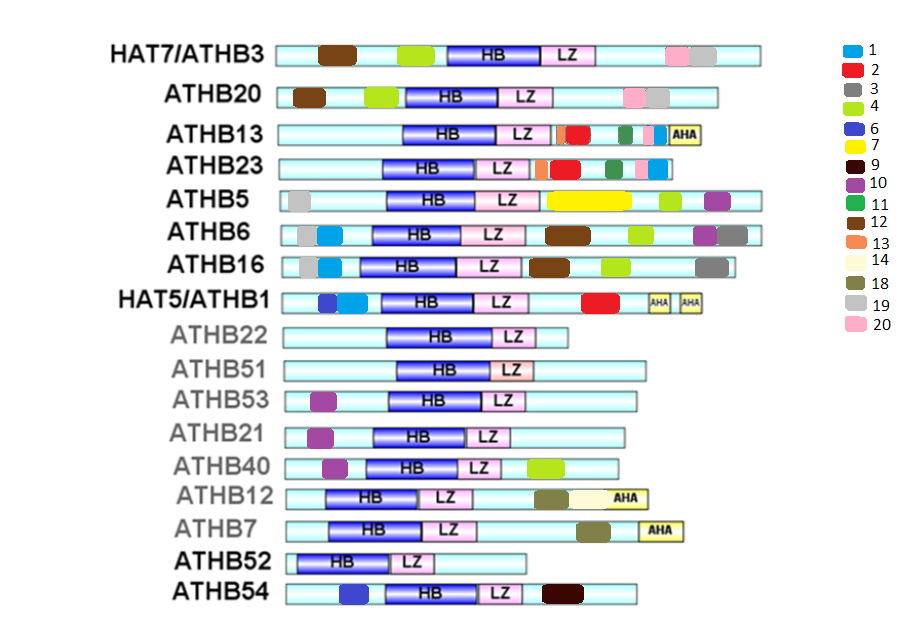


α

β2

ε

β1

δ

γ

ϕ1

ϕ2

C.


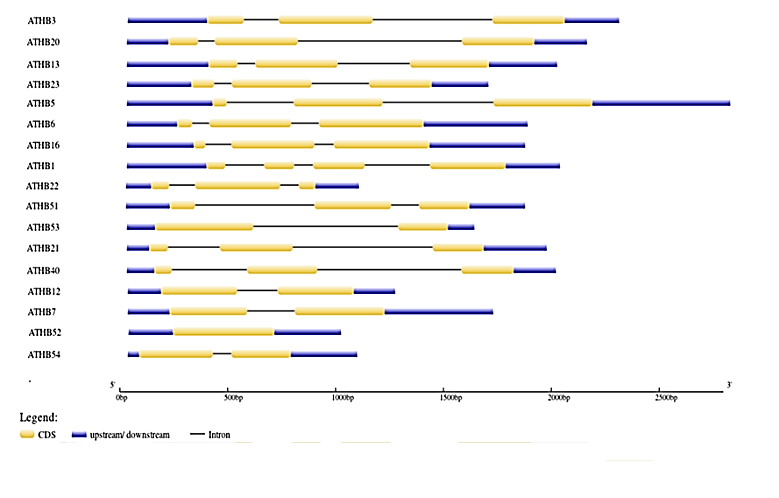

Supplement: Supplementary file 1 — Supplementary Fig. S1. The phylogenetic and structural characterization of the HD-Zip I subfamily in A. thaliana. A. The phylogenetic tree of Arabidopsis HD-Zip I subfamily based on alignment of full-length protein sequences by using MAGA 11, B. Structural domain organizations of Arabidopsis HD-Zip I proteins including the conserved domain (HD and Zip) and additional motifs. Uncharacterized motifs outside the HD and Zip as a potential source of functional diversity are numbered according to Arce et al. (2011). C. Schematic diagram representing the structure of Arabidopsis HD-Zip I genes. Yellow boxes represent exons and spaces between boxes correspond to introns. The 5’ and 3’ UTRs are marked by blue boxes. The exon/intron structure of each HD-Zip I gene was defined by comparison of their genomic and cDNA sequences. The sizes of exons, introns and UTRs are drawn to scale as indicated below. The gene structures were illustrated using the Gene Structure Display Server (http://gsds.cbi.pku.edu.cn/ (DOCX 295 KB) [file 239_2023_10121_MOESM1_ESM.docx]

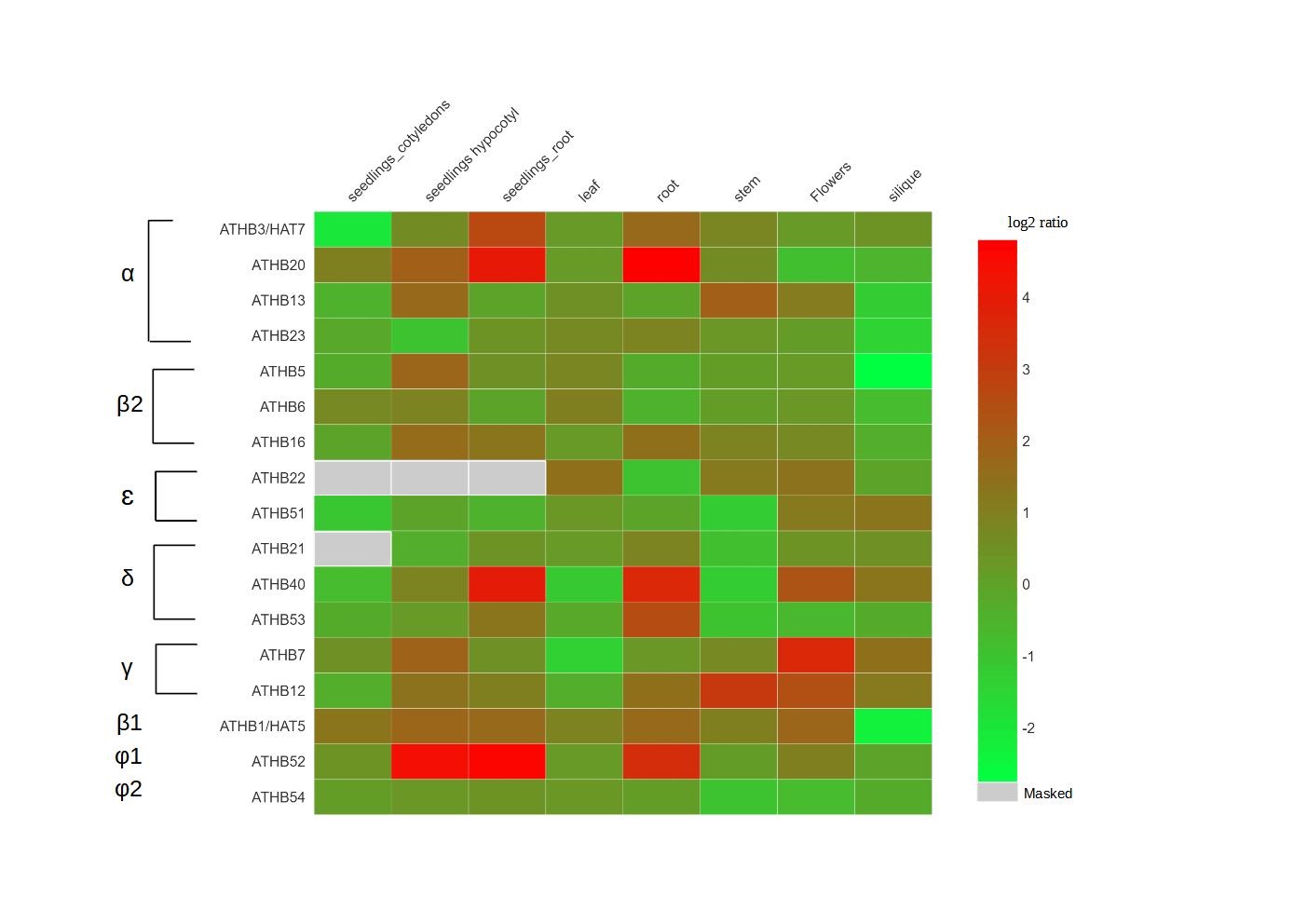

Supplement: Supplementary file 2 — Supplementary Fig. S2. Expression profiles of 17 HD-Zip I genes in different tissues and stages of development of A. thaliana. The data are based on Arabidopsis eFP Browser (The BAR and other Data Analysis Tools for Plant Biology (utoronto.ca). The heatmap shows the log2-transformed TPM values of each gene. The expression level of AtHB genes is represented using color scale ranging from green (low expression) to red (high expression (JPG 109 kb) [file 239_2023_10121_MOESM2_ESM.jpg]

## Slide 1
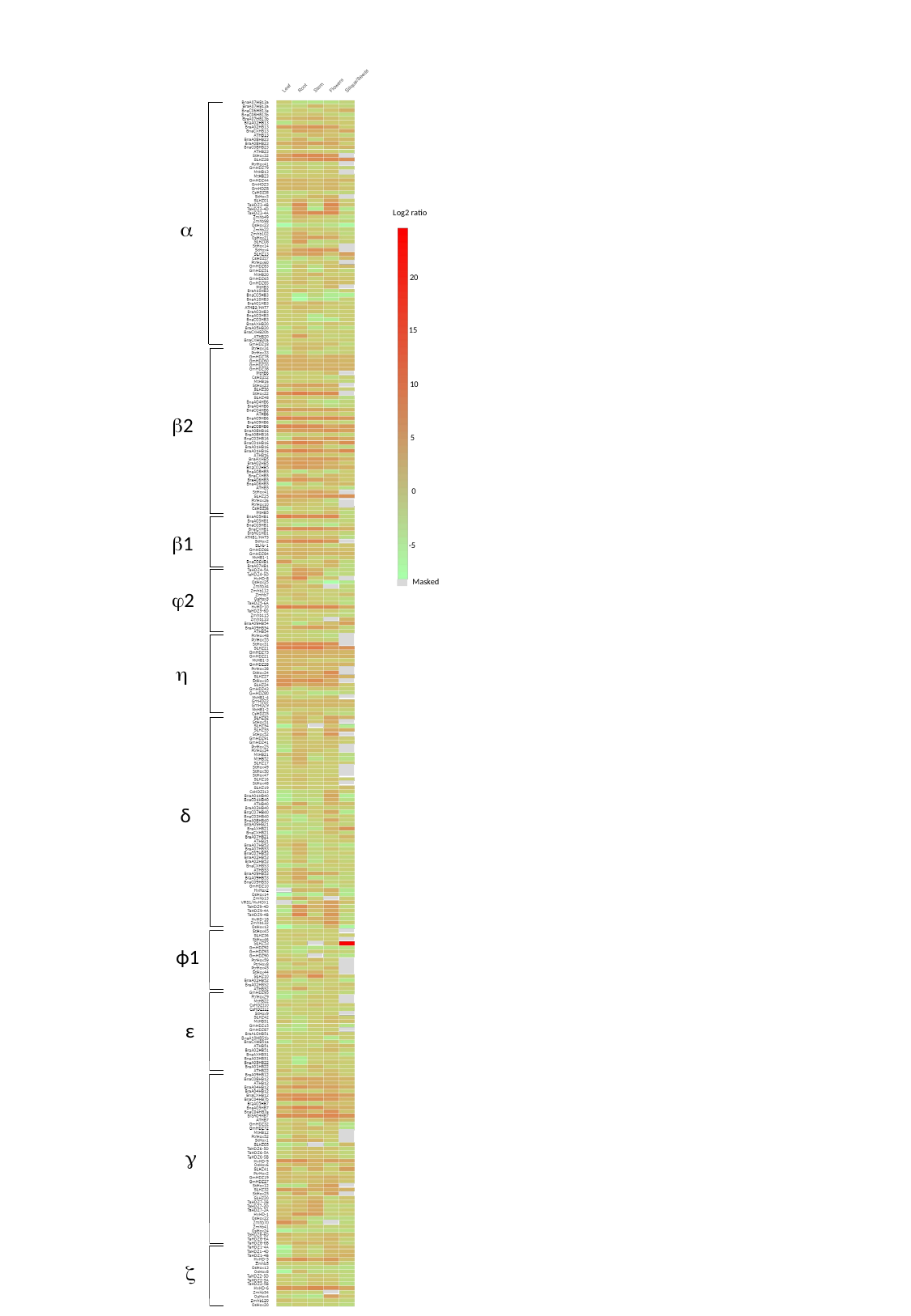

Log2 ratio
α
20
15
10
β2
5
0
β1
-5
2

δ
ϕ1
ε
γ

Masked

Supplement: Supplementary file 3 — Supplementary file3 Fig. S3. Expression profiles of HD-Zip I genes in five tissues of the selected plant species. The tissues include leaves, stem, roots, flowers and silique/seeds. The Reads/Kb/Million (RPKM) normalized values of expressed genes were log2-transformed and visualized as heatmap. The order of genes in the heatmap is consistency with the phylogeny in Figure 2. The data were from the different databases or the published studies. Data were obtained from: H. vulgare - Barley RNA-seq Database morexGenes - Barley Assembly and RNA-seq (hutton.ac.uk); G. max - soybean expression atlas (Soybean expression atlas | Home (uenf.br)); S. lycopersicum - Tomato BARePlant (ePlant (utoronto.ca)); L. tuberosum - the PGSC database (Spud DB (uga.edu)); Z. mays - Maize eFP Browser (Maize eFP Browser (utoronto.ca)); M. truncatula - Medicago eFP Browser (Medicago eFP Browser (utoronto.ca)); B. napus - BrassicaEDB - A Gene Expression Database for Brassica Crops (BrassicaEDB - A Gene Expression Database for Brassica Crops (biodb.org)); B. rapa - the Brassicaceae Database (BRAD)(BRAD (brassicadb.cn)); O. sativa - Rice eFP Browser (https://bar.utoronto.ca/efprice/cgi-bin/efpWeb.cgi) (PPTX 968 KB) [file 239_2023_10121_MOESM3_ESM.pptx]

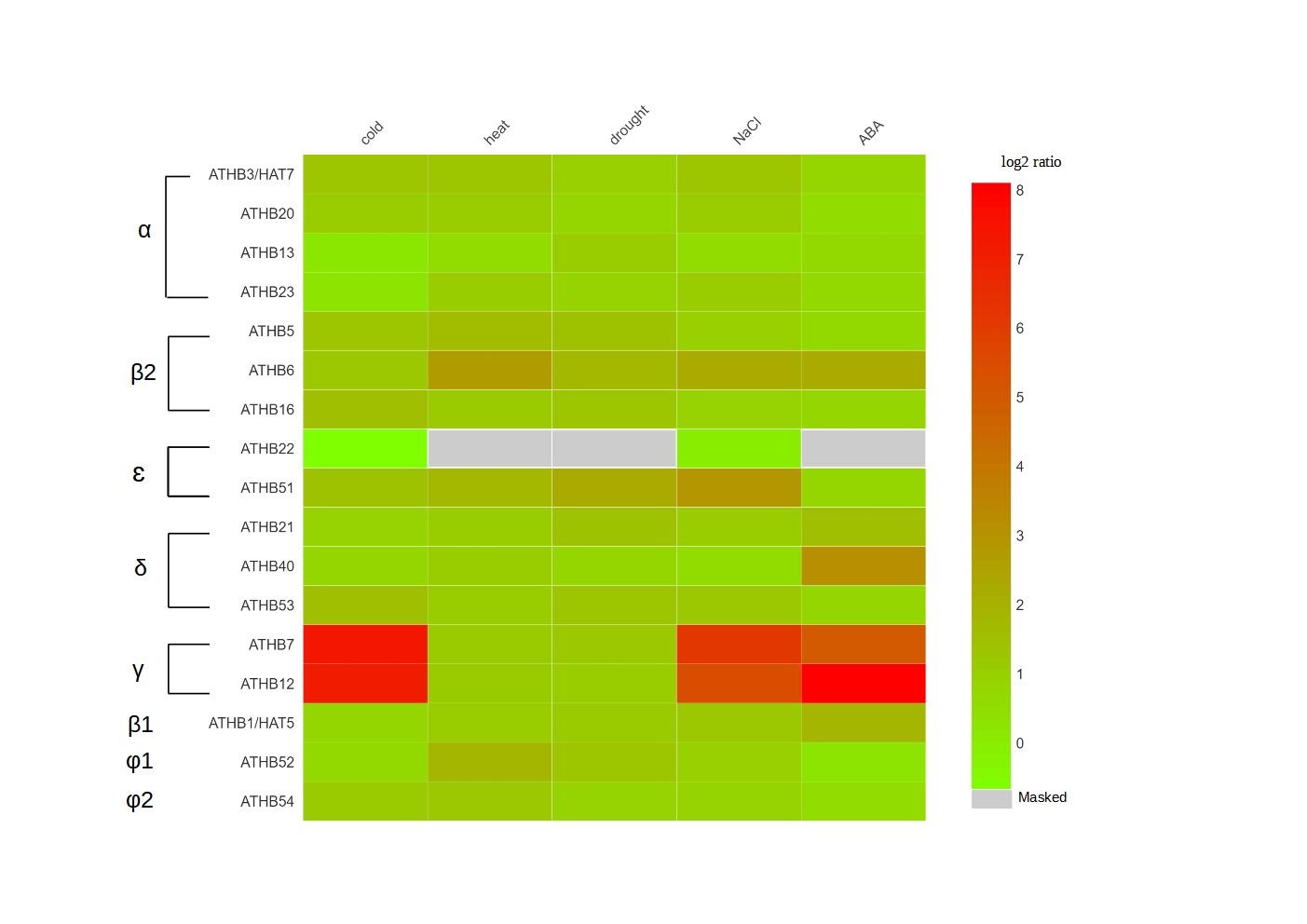

Supplement: Supplementary file 4 — Supplementary file4 Fig. S4. Expression profiles of HD-Zip I genes under abiotic stresses and ABA treatment of A. thaliana. The data are based on Arabidopsis eFP Browser (The BAR and other Data Analysis Tools for Plant Biology (utoronto.ca). The expression level are based on the log2 values and color scale represent the expression levels of AtHB genes from green (downregulated) to red (upregulated) (JPG 99 kb) [file 239_2023_10121_MOESM4_ESM.jpg]

## Slide 1
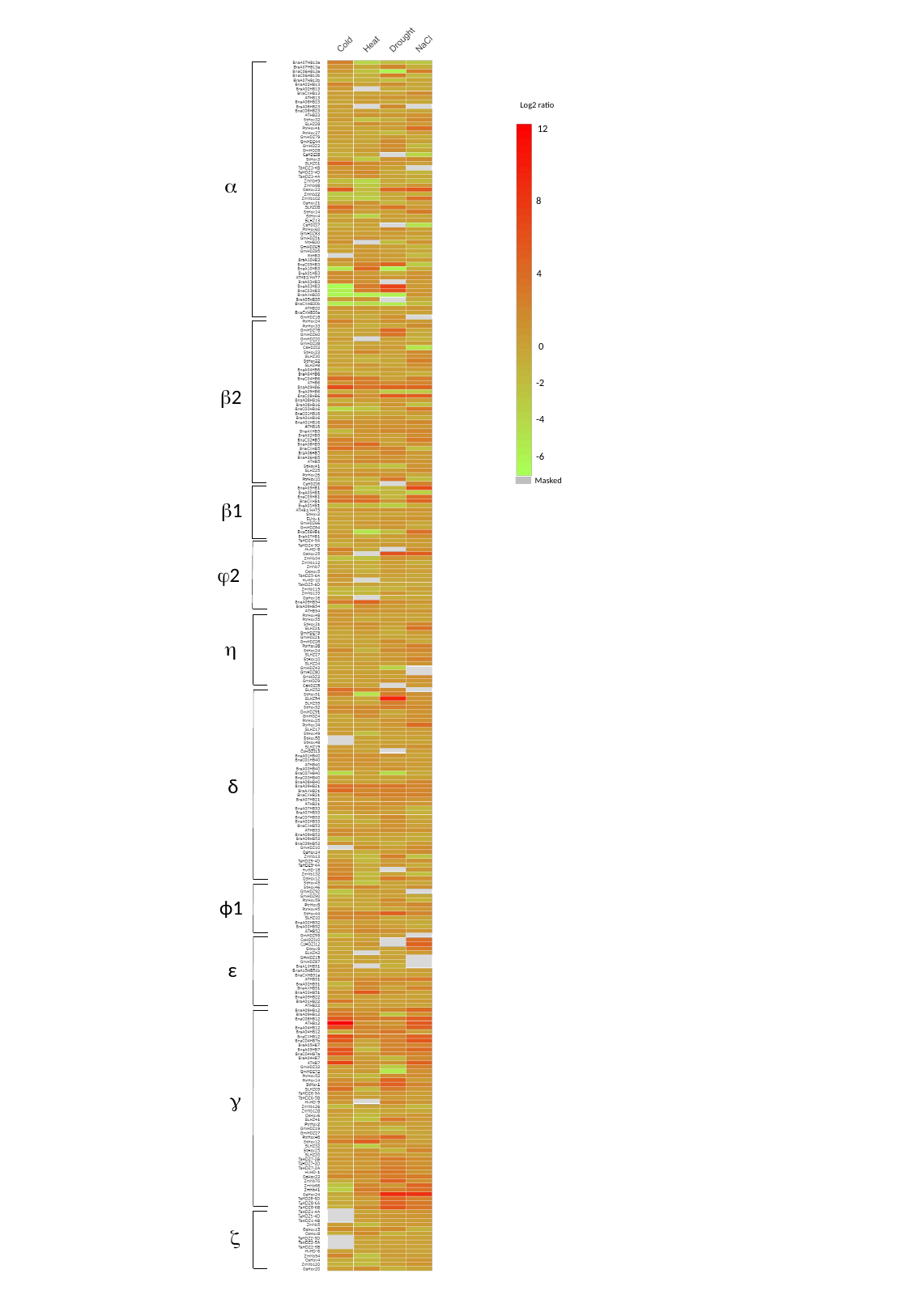

12
8
4
0
-2
-4
-6
Log2 ratio
α
β2
β1
2

δ
ϕ1
ε
γ

Masked

Supplement: Supplementary file 5 — Supplementary file5 Fig S5. Expression profiles of HD-Zip I genes in response to cold, heat, drought and salt stresses in different species. The Reads/Kb/Million (RPKM) normalized values of expressed genes was log2-transformed and visualized as heatmap. The order of genes in the heatmap is consistency with the phylogeny in Figure 2. Data were obtained from: B. napus - BrassicaEDB - A Gene Expression Database for Brassica Crops (BrassicaEDB - A Gene Expression Database for Brassica Crops (biodb.org)); B. rapa - based on data from Khan et al. (2018); G. max obtained from the NCBI GEO database under accession numbers GSE40627 (drought), GSE41125 (salinity) and GSE213479 (heat); H. vulgare: obtained from the NCBI GEO database under accession numbers PRJEB18276 (cold), PRJNA324116 (heat), PRJNA439267 (drought) and PRJEB13621 (salt); S. lycopersicum - Tomato BARePlant (ePlant (utoronto.ca)) and obtained from the NCBI GEO database under accession number PRJNA730730 (heat); L. tuberosum - the PGSC database (Spud DB (uga.edu)); Z. mays - Maize eFP Browser (Maize eFP Browser (utoronto.ca)); M. truncatula - based on data from Li et al. (2022); P. trichocarpa - Poplar eFP Browser https://bar.utoronto.ca/eplant_poplar/; C. sativa - Cucurbit Genomics Database: Cucurbit Genomics Database (CuGenDB); O. sativa - Rice eFP Browser (https://bar.utoronto.ca/efprice/cgi-bin/efpWeb.cgi) (PPTX 186 KB) [file 239_2023_10121_MOESM5_ESM.pptx]
