# Supplementary material for: Evolutionary Consequences of Functional and Regulatory Divergence of HD-Zip I Transcription Factors as a Source of Diversity in Protein Interaction Networks in Plants
Source: J Mol Evol. 2023 Jun 23;91(5):581–97. doi: 10.1007/s00239-023-10121-4 (PMC10598176; doi:10.1007/s00239-023-10121-4)
Supplement: Supplementary file 6 — Supplementary file6 Table S1. Number of transcription factors (TFs) in plant genomes based on data derived from PlantTFDB v5.0 (http://planttfdb.gao-lab.org/) and PLAZA project (https://bioinformatics.psb.ugent.be/plaza/) (DOCX 35 KB) [file 239_2023_10121_MOESM6_ESM.docx]

Table S1.

| Species | Number of TF | Number of TF families | Number of genes | % of total genes | Reference |
| --- | --- | --- | --- | --- | --- |
| *Amaranthus hypochondriacus* | 1259 | 56 | 23,847 | 5.2 | PLAZA project |
| *Aquilegia coerulea* | 2095 | 58 | 29,550 | 7.0 | Filiault et al. 2018 |
| *Actinidia chinensis* | 2296 | 58 | 39,040 | 5.9 | PLAZA project |
| *Artemisia annua* | 625 | 49 | 63 226 | 1.0 | Shen et al. 2018 |
| *Capsicum annuum* | 1665 | 58 | 35,884 | 4.6 | PLAZA project |
| *Catharanthus roseus* | 2405 | 55 | 33,829 | 7.1 | She et al. 2019 |
| *Coffea canephora* | 1256 | 57 | 25,574 | 4.9 | PLAZA project |
| *Daucus carota* | 1906 | 56 | 32,113 | 5.9 | PLAZA project |
| *Dorcoceras hygrometricum* | 1342 | 56 | 48,040 | 2.8 | NCBI |
| *Genlisea aurea* | 966 | 57 | 17,755 | 5.4 | Leushkin et al. 2013 |
| *Helianthus annuus* | 288 | 46 | 57,237 | 0.5 | NCBI |
| *Ipomoea trifida* | 2069 | 57 | 32,301 | 6.4 | NCBI |
| *Lactuca sativa* | 1036 | 55 | 38,910 | 2.7 | Phytozome |
| *Mimulus guttatus* | 2016 | 57 | 28,140 | 7.2 | Phytozome |
| *Nicotiana benthamiana* | 3206 | 57 | 53,411 | 6.0 | Kourelis et al. 2019 |
| *Nicotiana sylvestris* | 2807 | 57 | 33,816 | 8.3 | Sierro et al. 2013 |
| *Nicotiana tabacum* | 5176 | 57 | 61,780 | 8.4 | Sugiyama et al. 2005 |
| *Nicotiana tomentosiformis* | 2898 | 58 | 30,887 | 9.4 | NCBI |
| *Ocimum tenuiflorum* | 1723 | 56 | 36,768 | 4.7 | Upadhyay et al. 2015 |
| *Petunia axillaris* | 1999 | 57 | 32,928 | 6.1 | Bombarely et al. 2016 |
| *Petunia inflata* | 2099 | 57 | 36,697 | 5.7 | Bombarely et al. 2016 |
| *Salvia miltiorrhiza* | 1621 | 56 | 32,483 | 5.0 | Song et al. 2020 |
| *Sesamum indicum* | 2326 | 58 | 27,148 | 8.6 | Wang et al. 2014 |
| *Solanum lycopersicum* | 1845 | 58 | 34,725 | 5.3 | PLAZA project |
| *Solanum melongena* | 1586 | 57 | 85,446 | 1.9 | Hirakawa et al. 2014 |
| *Solanum pennellii* | 1971 | 58 | 26,874 | 7.3 | NCBI |
| *Solanum pimpinellifolium* | 1770 | 58 | 25,970 | 6.8 | Rozali et al. 2018 |
| *Solanum tuberosum* | 2405 | 56 | 39,028 | 6.2 | PLAZA project |
| *Utricularia gibba* | 1672 | 55 | 25,930 | 6.4 | PLAZA project |
| *Beta vulgaris* | 1163 | 57 | 26,920 | 4.3 | PLAZA project |
| *Dianthus caryophyllus* | 1318 | 57 | 43 266 | 3.0 | Yagi et al. 2013 |
| *Arachis duranensis* | 1828 | 58 | 34,553 | 5.3 | NCBI |
| *Arachis hypogaea* | 799 | 52 | 41,840 | 1.9 | PLAZA project |
| *Arachis ipaensis* | 1946 | 57 | 37,716 | 5.2 | NCBI |
| *Cajanus cajan* | 1886 | 56 | 48,680 | 3.9 | PLAZA project |
| *Cannabis sativa* | 1225 | 56 | 30,074 | 4.1 | van Bakel et al. 2011 |
| *Castanea mollissima* | 1499 | 57 | 36,479 | 4.1 | Xing et al. 2019 |
| *Cicer arietinum* | 2235 | 58 | 23,550 | 9.5 | PLAZA project |
| *Citrullus lanatus* | 1355 | 58 | 23,440 | 5.8 | PLAZA project |
| *Cucumis melo* | 1537 | 58 | 27,427 | 5.6 | PLAZA project |
| *Cucumis sativus* | 1931 | 57 | 21,503 | 9.0 | PLAZA project |
| *Fragaria vesca* | 1485 | 58 | 32,831 | 4.5 | PLAZA project |
| *Fragaria x ananassa* | 1250 | 58 | 30,703 | 4.1 | Edger et al. 2019 |
| *Glycine max* | 6150 | 57 | 56,044 | 11.0 | PLAZA project |
| *Glycine soja* | 3193 | 56 | 47,201 | 6.8 | NCBI |
| *Humulus lupulus* | 1324 | 56 | 41,228 | 3.2 | Natsume et al. 2014 |
| *Jatropha curcas* | 1467 | 57 | 19,420 | 7.6 | NCBI |
| *Juglans regia* | 1591 | 54 | 32 498 | 4.9 | Martínez‐García et al. 2016 |
| *Linum usitatissimum* | 2481 | 57 | 43,471 | 5.7 | Phytozome |
| *Lotus japonicus* | 2056 | 56 | 30,799 | 6.7 | Sato et al. 2008 |
| *Malus domestica* | 3119 | 58 | 57,386 | 5.4 | Velasco et al. 2010 |
| *Manihot esculenta* | 2676 | 57 | 33,033 | 8.1 | Phytozome |
| *Medicago truncatula* | 2741 | 57 | 50,894 | 5.4 | PLAZA project |
| *Morus notabilis* | 1289 | 58 | 21,636 | 4.7 | NCBI |
| *Phaseolus vulgaris* | 2177 | 58 | 27,433 | 7.9 | Phytozome |
| *Populus euphratica* | 2382 | 58 | 30,684 | 7.8 | NCBI |
| *Populus trichocarpa* | 4287 | 58 | 42,950 | 10.0 | PLAZA project |
| *Prunus mume* | 1893 | 58 | 23,946 | 7.9 | NCBI |
| *Prunus persica* | 2780 | 58 | 26,873 | 10.3 | PLAZA project |
| *Pyrus bretschneideri* | 2353 | 57 | 42,812 | 5.5 | PLAZA project |
| *Ricinus communis* | 1299 | 57 | 31,221 | 4.2 | PLAZA project |
| *Salix purpurea* | 4412 | 58 | 37,865 | 11.7 | Phytozome |
| *Trifolium pratense* | 2065 | 46 | 39,948 | 5.2 | PLAZA project |
| *Vigna angularis* | 2233 | 56 | 34,183 | 6.5 | Kang et al. 2015 |
| *Vigna radiata* | 1539 | 56 | 22,368 | 6.9 | PLAZA project |
| *Vigna unguiculata* | 488 | 48 | 29,773 | 1.6 | Phytozome |
| *Ziziphus jujuba* | 2152 | 58 | 29,293 | 7.3 | PLAZA project |
| *Kalanchoe laxiflora* | 2926 | 71 | 50,461 | 5.8 | Phytozome |
| *Aethionema arabicum* | 1371 | 58 | 23,160 | 5.9 | Fernandez-Pozo et al. 2021 |
| *Arabidopsis halleri* | 1586 | 57 | 25,008 | 6.3 | Phytozome |
| *Arabidopsis lyrata* | 1759 | 58 | 31,073 | 5.7 | PLAZA project |
| *Arabidopsis thaliana* | 2296 | 58 | 35,386 | 6.5 | TAIR10 |
| *Arabis alpina* | 1215 | 56 | 21,609 | 5.6 | Willing et al. 2015 |
| *Azadirachta indica* | 1900 | 58 | ~20,000 | 9.5 | Krishnan et al. 2012 |
| *Boechera stricta* | 1907 | 58 | 27,416 | 7.0 | Phytozome |
| *Brassica napus* | 5985 | 58 | 101,040 | 5.9 | Chalhoub et al. 2014 |
| *Brassica oleracea* | 4272 | 58 | 59,220 | 7.2 | PLAZA project |
| *Brassica rapa* | 4127 | 58 | 40,492 | 10.2 | PLAZA project |
| *Camelina sativa* | 5405 | 58 | 81,485 | 6.6 | NCBI |
| *Capsella grandiflora* | 1784 | 58 | 24,805 | 7.2 | Phytozome |
| *Capsella rubella* | 1898 | 58 | 26,521 | 7.2 | PLAZA project |
| *Carica papaya* | 1379 | 58 | 28,629 | 4.8 | Ming et al. 2008 |
| *Citrus clementina* | 1905 | 58 | 24,533 | 7.8 | PLAZA project |
| *Citrus sinensis* | 2255 | 58 | 25,376 | 8.9 | Phytozome |
| *Eucalyptus camaldulensis* | 1937 | 56 | 77,121 | 2.5 | Hirakawa et al. 2011 |
| *Eucalyptus grandis* | 2163 | 56 | 36,349 | 6.0 | PLAZA project |
| *Eutrema salsugineum* | 1892 | 58 | 26,351 | 7.2 | Phytozome |
| *Gossypium arboreum* | 2532 | 58 | 34,410 | 7.4 | NCBI |
| *Gossypium hirsutum* | 5022 | 58 | 66,577 | 7.5 | Phytozome |
| *Gossypium raimondii* | 4894 | 58 | 37,505 | 13.0 | PLAZA project |
| *Raphanus raphanistrum* | 2734 | 58 | 32,670 | 8.4 | Moghe et al. 2014 |
| *Raphanus sativus* | 3010 | 58 | 61,572 | 4.9 | Kitashiba et al. 2014 |
| *Sisymbrium irio* | 1710 | 58 | 28,917 | 5.9 | Haudry et al. 2013 |
| *Tarenaya hassleriana* | 3311 | 88 | 27,396 | 12.1 | PLAZA project |
| *Thellungiella parvula* | 1672 | 58 | 28,457 | 5.9 | Wu 2012 |
| *Theobroma cacao* | 2224 | 58 | 29,232 | 7.6 | PLAZA project |
| *Nelumbo nucifera* | 1476 | 57 | 26,685 | 5.5 | PLAZA project |
| *Spinacia oleracea* | 1082 | 57 | 25,495 | 4.2 | Xu et al. 2017 |
| *Vitis vinifera* | 1276 | 58 | 26,346 | 4.8 | PLAZA project |
| *Aegilops tauschii* | 1439 | 55 | 39,622 | 3.6 | Luo et al. 2017 |
| *Ananas comosus* | 1277 | 57 | 27,024 | 4.7 | Phytozome |
| *Brachypodium distachyon* | 2898 | 56 | 34,310 | 8.4 | Phytozome |
| *Brachypodium stacei* | 2121 | 56 | 29,898 | 7.1 | Phytozome |
| *Dichanthelium oligosanthes* | 1657 | 54 | 30,153 | 5.5 | Studer, 2016 |
| *Elaeis guineensis* | 2910 | 57 | 34,800 | 8.4 | Singh et al. 2013 |
| *Eragrostis tef* | 2090 | 55 | 38,000 | 5.5 | Phytozome |
| *Hordeum vulgare* | 2620 | 56 | 39,734 | 6.6 | Phytozome |
| *Leersia perrieri* | 2024 | 55 | 29,078 | 7.0 | EnsemblPlants |
| *Musa acuminata* | 2896 | 57 | 36,528 | 7.9 | Phytozome |
| *Oropetium thomaeum* | 1290 | 54 | 28,446 | 4.5 | Phytozome |
| *Oryza barthii* | 1775 | 55 | 34,575 | 5.1 | ENA, Oryza Genome Evolution Project |
| *Oryza brachyantha* | 1444 | 56 | 32,038 | 4.5 | Chen et al. 2013 |
| *Oryza glaberrima* | 1579 | 56 | 33,164 | 4.8 | ENA, Oryza Genome Evolution Project |
| *Oryza glumaepatula* | 2029 | 55 | 33,069 | 6.1 | Szareski et al. 2018 |
| *Oryza longistaminata* | 1223 | 54 | 34,389 | 3.6 | Reuscher et al. 2018 |
| *Oryza meridionalis* | 1949 | 56 | 21,169 | 9.2 | Brozynska M 2016 |
| *Oryza nivara* | 2147 | 56 | 36,313 | 5.9 | ENA, Oryza Genome Evolution Project |
| *Oryza punctata* | 2019 | 56 | 31,762 | 6.4 | ENA, Oryza Genome Evolution Project |
| *Oryza rufipogon* | 2032 | 55 | 37,071 | 5.5 | Huang et al. 2012 |
| *Oryza sativa subsp. indica* | 1891 | 56 | 37,344 | 5.1 | UniProt |
| *Oryza sativa subsp. japonica* | 2408 | 56 | 42,189 | 5.7 | PLAZA project |
| *Panicum hallii* | 2528 | 56 | 37,232 | 6.8 | Phytozome |
| *Panicum virgatum* | 5702 | 56 | 98,007 | 5.8 | Phytozome |
| *Phalaenopsis equestris* | 1420 | 57 | 29,431 | 4.8 | Cai et al. 2014 |
| *Phoenix dactylifera* | 1426 | 56 | 28,800 | 5.0 | Al-Dous et al. 2011 |
| *Phyllostachys heterocycla* | 1768 | 54 | 31,987 | 5.5 | Peng et al. 2013 |
| *Saccharum officinarum* | 672 | 48 |  |  | Phytozome |
| *Setaria italica* | 2410 | 56 | 34,584 | 7.0 | Phytozome |
| *Setaria viridis* | 2765 | 56 | 38,334 | 7.2 | Phytozome |
| *Sorghum bicolor* | 2654 | 56 | 34,129 | 7.8 | Phytozome |
| *Spirodela polyrhiza* | 1046 | 57 | 19,623 | 5.3 | Phytozome |
| *Triticum aestivum* | 3606 | 56 | 99,386 | 3.6 | Phytozome |
| *Triticum urartu* | 1238 | 52 | 41,507 | 3.0 | Ling et al. 2018 |
| *Zea mays* | 3308 | 56 | 39,498 | 8.4 | PLAZA project |
| *Zostera marina* | 1271 | 56 | 20,450 | 6.2 | Olsen et al. 2016 |
| *Zoysia japonica* | 2299 | 53 | 59,271 | 3.9 | Tanaka et al. 2016 |
| *Zoysia matrella* | 3955 | 54 | 95,079 | 4.2 | Tanaka et al. 2016 |
| *Zoysia pacifica* | 2604 | 55 | 65,252 | 4.0 | Tanaka et al. 2016 |
| *Picea abies* | 1107 | 54 | 66,632 | 1.7 | PLAZA project |
| *Pinus taeda* | 442 | 47 | 9,024 | 4.9 | Stevens et al. 2016 |
| *Pseudotsuga menziesii* | 1915 | 54 | 54,830 | 3.5 | Neale et al. 2017 |

References:

Al-Dous E, George B, Al-Mahmoud M et al (2011) De novo genome sequencing and comparative genomics of date palm (*Phoenix dactylifera*). Nat Biotechnol 29:521-527. <https://doi.org/10.1038/nbt.1860>

van Bakel H, Stout JM, Cote AG et al (2011) The draft genome and transcriptome of *Cannabis sativa*. Genome Biol 12:R102 <https://doi.org/10.1186/gb-2011-12-10-r102>

Bombarely A, Moser M, Amrad A et al (2016) Insight into the evolution of the Solanaceae from the parental genomes of *Petunia* hybrida. Nature Plants 2:16074. <https://doi.org/10.1038/nplants.2016.74>

Brozynska M, Copetti D, Furtado A et al (2017) Sequencing of Australian wild rice genomes reveals ancestral relationships with domesticated rice. Plant Biotechnol J 15:765-774. <https://doi.org/10.1111/pbi.12674>

Cai J, Liu X, Vanneste K et al (2015) The genome sequence of the orchid *Phalaenopsis equestris*. Nat Genet 47:65-72. <https://doi.org/10.1038/ng.3149>

Chalhoub B, Denoeud F, Liu S et al (2014) Early allopolyploid evolution in the post-Neolithic *Brassica napus* oilseed genome. Science 345:950-953. <https://doi.org/10.1126/science.1253435>

Chen J, Huang Q, Gao D et al (2013) Whole-genome sequencing of *Oryza brachyantha* reveals mechanisms underlying *Oryza* genome evolution. Nat Commun 4:1595. <https://doi.org/10.1038/ncomms2596>

Edger PP, Poorten TJ, VanBuren R et al (2019) Origin and evolution of the octoploid strawberry genome. Nat Genet 51:541-547. <https://doi.org/10.1038/s41588-019-0356-4>

Fernandez-Pozo N, Metz T, Chandler JO et al (2021) *Aethionema arabicum* genome annotation using PacBio full-length transcripts provides a valuable resource for seed dormancy and Brassicaceae evolution research. Plant J 106:275-293. <https://doi.org/10.1111/tpj.15161>

Filiault DL, Ballerini ES, Mandáková T et al (2018) The *Aquilegia* genome provides insight into adaptive radiation and reveals an extraordinarily polymorphic chromosome with a unique history. Elife 7:e36426. <https://doi.org/10.7554/eLife.36426>

Haudry A, Platts A, Vello E et al (2013) An atlas of over 90,000 conserved noncoding sequences provides insight into crucifer regulatory regions. Nat Genet 45:891-898. <https://doi.org/10.1038/ng.2684>

Hirakawa H, Shirasawa K, Miyatake K et al (2014) Draft genome sequence of eggplant (*Solanum melongena L.*): the representative *Solanum* species indigenous to the old world. DNA Res 21:649-660. <https://doi.org/10.1093/dnares/dsu027>

Hirakawa H, Nakamura Y, Kaneko T et al (2011) Survey of the genetic information carried in the genome of *Eucalyptus camaldulensis.* Plant Biotech 28:471-480. <https://doi.org/10.5511/plantbiotechnology.11.1027b>

Huang X, Kurata N, Wei X et al (2012) A map of rice genome variation reveals the origin of cultivated rice. Nature 490:497-501. <https://doi.org/10.1038/nature11532>

Kang Y, Satyawan D, Shim S et al (2015) Genome sequencing of adzuki bean (*Vigna angularis*) provides insight into high starch and low fat accumulation and domestication. Proc Natl Acad Sci USA 112:13213-13218. <https://doi.org/10.1073/pnas.1420949112>

Kitashiba H, Li F, Hirakawa H et al (2014) Draft sequences of the radish (*Raphanus sativus L.*) genome. DNA Res 21:481-490. <https://doi.org/10.1093/dnares/dsu014>

Kourelis J, Kaschani, F, Grosse-Holz FM et al (2019) A homology-guided, genome-based proteome for improved proteomics in the alloploid *Nicotiana benthamiana*. BMC Genomics 20:722. <https://doi.org/10.1186/s12864-019-6058-6>

Krishnan NM, Pattnaik S, Jain P et al (2012) A draft of the genome and four transcriptomes of a medicinal and pesticidal angiosperm *Azadirachta indica*. BMC Genomics 13:464. <https://doi.org/10.1186/1471-2164-13-464>

Leushkin EV, Sutormin RA, Nabieva ER et al (2013) The miniature genome of a carnivorous plant *Genlisea aurea* contains a low number of genes and short non-coding sequences. BMC Genomics 14: 476. <https://doi.org/10.1186/1471-2164-14-476>

Ling HQ, Ma B, Shi X et al (2018) Genome sequence of the progenitor of wheat A subgenome *Triticum urartu*. Nature 557:424-428. <https://doi.org/10.1038/s41586-018-0108-0>

Luo MC, Gu Y, Puiu D et al (2017) Genome sequence of the progenitor of the wheat D genome *Aegilops tauschii*. Nature 551:498-502. <https://doi.org/10.1038/nature24486>

Martínez-García PJ, Crepeau MW, Puiu D et al (2016) The walnut (*Juglans regia*) genome sequence reveals diversity in genes coding for the biosynthesis of non-structural polyphenols. Plant J 87:507-532. <https://doi.org/10.1111/tpj.13207>

Ming R, Hou S, Feng Y et al (2008) The draft genome of the transgenic tropical fruit tree papaya (*Carica papaya Linnaeus*). Nature 452:991-996. <https://doi.org/10.1038/nature06856>

Moghe GD, Hufnagel DE, Tang H et al (2014) Consequences of whole-genome triplication as revealed by comparative genomic analyses of the wild Radish *Raphanus raphanistrum* and three other *Brassicaceae* species. Plant Cell 26:1925-1937. <https://doi.org/10.1105/tpc.114.124297>

Natsume S, Takagi H, Shiraishi A et al (2015) The draft genome of hop (*Humulus lupulus*), an essence for brewing. Plant Cell Physiol 56:428-441. <https://doi.org/10.1093/pcp/pcu169>

Neale DB, McGuire PE, Wheeler NC et al (2017) The douglas-fir genome sequence reveals specialization of the photosynthetic apparatus in *Pinaceae*. G3 (Bethesda) 7:3157-3167. <https://doi.org/10.1534/g3.117.300078>

Olsen J, Rouzé P, Verhelst B et al (2016) The genome of the seagrass *Zostera marina* reveals angiosperm adaptation to the sea. Nature 530:331-335. <https://doi.org/10.1038/nature16548>

Peng Z, Lu Y, Li L et al (2013) The draft genome of the fast-growing non-timber forest species moso bamboo (*Phyllostachys heterocycla*). Nat Genet 45:456–461. <https://doi.org/10.1038/ng.2569>

Reuscher S, Furuta T, Bessho-Uehara K et al (2018) Assembling the genome of the African wild rice *Oryza longistaminata* by exploiting synteny in closely related *Oryza* species. Commun Biol 1:162. <https://doi.org/10.1038/s42003-018-0171-y>

Sato S, Nakamura Y, Kaneko T et al (2008) Genome Structure of the Legume, *Lotus japonicus*. DNA Res 15:227-239. <https://doi.org/10.1093/dnares/dsn008>

She J, Yan H, Yang J, Xu W, Su Z (2019) croFGD: *Catharanthus roseus* functional genomics database. Front Genetic 10:238. <https://doi.org/10.3389/fgene.2019.00238>

Shen XX, Opulente DA, Kominek J et al (2018) Tempo and mode of genome evolution in the budding yeast subphylum. Cell 175:1533-1545. <https://doi.org/10.1016/j.cell.2018.10.023>

Sierro N, Battey JN, Ouadi S et al (2013) Reference genomes and transcriptomes of *Nicotiana sylvestris* and *Nicotiana tomentosiformis*. Genome Biol 14:R60. <https://doi.org/10.1186/gb-2013-14-6-r60>

Singh R, Ong-Abdullah M, Low ET et al (2013) Oil palm genome sequence reveals divergence of interfertile species in Old and New worlds. Nature 500:335-339. <https://doi.org/10.1038/nature12309>

Song Z, Lin C, Xing P et al (2020) A high-quality reference genome sequence of *Salvia miltiorrhiza* provides insights into tanshinone synthesis in its red rhizomes. Plant Genome 13:e20041. <https://doi.org/10.1002/tpg2.20041>

Sugiyama Y, Watase Y, Nagase M et al (2005) The complete nucleotide sequence and multipartite organization of the tobacco mitochondrial genome: comparative analysis of mitochondrial genomes in higher plants. Mol Genet Genomics 272:603-615. <https://doi.org/10.1007/s00438-004-1075-8>

Stevens KA (2016) Sequence of the sugar pine megagenome. Genetics 204:1613-1626. <https://doi.org/10.1534/genetics.116.193227>

Studer AJ, Schnable JC, Weissmann S et al (2016) The draft genome of the C3 panicoid grass species *Dichanthelium oligosanthes*. Genome Biol 17:223. <https://doi.org/10.1186/s13059-016-1080-3>

Szareski VJ, Carvalho IR, Rosa TC et al (2018) *Oryza* wild species: An alternative for rice breeding under abiotic stress conditions. Amer J Plant Sci 9:84416. <https://doi.org/10.4236/ajps.2018.96083>

Upadhyay S, Dixit M (2015) Role of polyphenols and other phytochemicals on molecular signaling. Oxid Med and Cell Longev 2015:504253. <https://doi.org/10.1155/2015/504253>

Tanaka H, Hirakawa H, Kosugi S et al (2016) Sequencing and comparative analyses of the genomes of zoysia grasses. DNA Res 23:171–180. <https://doi.org/10.1093/dnares/dsw006>

Velasco R, Zharkikh A, Affourtit J et al (2010) The genome of the domesticated apple (*Malus × domestica Borkh*.). Nat Genet 42:833-839. <https://doi.org/10.1038/ng.654>

Wang L, Yu S, Tong C et al (2014) Genome sequencing of the high oil crop sesame provides insight into oil biosynthesis. Genome Biol 15:R39. <https://doi.org/10.1186/gb-2014-15-2-r39>

Willing EM, Rawat V, Mandáková T et al (2015) Genome expansion of *Arabis alpina* linked with retrotransposition and reduced symmetric DNA methylation. Nature Plants 1:14023. <https://doi.org/10.1038/nplants.2014.23>

Wu HJ, Zhang Z, Wang JY et al (2012) Insights into salt tolerance from the genome of *Thellungiella salsuginea*. Proc Nat Acad Sci USA 109:12219-12224. <https://doi.org/10.1073/pnas.1209954109>

Xing Y, Yang L, Qing Z et al (2019) Hybrid de novo genome assembly of Chinese chestnut (*Castanea mollissima*). GigaScience 8:9. <https://doi.org/10.1093/gigascience/giz112>

Xu C, Jiao C, Sun H et al (2017) Draft genome of spinach and transcriptome diversity of 120 *Spinacia* accessions. Nat Commun 8:15275. <https://doi.org/10.1038/ncomms15275>

Yagi M, Kosugi S, Hirakawa H et al (2014) Sequence analysis of the genome of carnation (*Dianthus caryophyllus* L.). DNA Res 21:231-241. <https://doi.org/10.1093/dnares/dst053>
