# Supplementary material for: Evolutionary Consequences of Functional and Regulatory Divergence of HD-Zip I Transcription Factors as a Source of Diversity in Protein Interaction Networks in Plants
Source: J Mol Evol. 2023 Jun 23;91(5):581–97. doi: 10.1007/s00239-023-10121-4 (PMC10598176; doi:10.1007/s00239-023-10121-4)
Supplement: Supplementary file 7 — Supplementary Table S2. Numbers of HD-Zip genes in plant genomes (DOCX 16 KB) [file 239_2023_10121_MOESM7_ESM.docx]

Table S2

| **Species** | **HD-Zip** |  | **Species** | **HD-Zip** |
| --- | --- | --- | --- | --- |
| *Helianthus annuus* | 15 |  | *Azadirachta indica* | 51 |
| *Pinus taeda* | 16 |  | *Thellungiella parvula* | 51 |
| *Artemisia annua* | 17 |  | *Boechera stricta* | 52 |
| *Vigna unguiculata* | 17 |  | *Ipomoea trifida* | 52 |
| *Saccharum officinarum* | 18 |  | *Daucus carota* | 52 |
| *Eucalyptus camaldulensis* | 23 |  | *Oryza meridionalis* | 52 |
| *Spirodela polyrhiza* | 23 |  | *Eucalyptus grandis* | 52 |
| *Triticum urartu* | 24 |  | *Eutrema salsugineum* | 52 |
| *Fragaria x ananassa* | 24 |  | *Cajanus cajan* | 53 |
| *Spinacia oleracea* | 24 |  | *Capsella rubella* | 53 |
| *Oryza longistaminata* | 24 |  | *Leersia perrieri* | 54 |
| *Arachis hypogaea* | 25 |  | *Arabidopsis halleri* | 55 |
| *Beta vulgaris* | 27 |  | *Pyrus bretschneideri* | 56 |
| *Fragaria vesca* | 28 |  | *Theobroma cacao* | 56 |
| *Carica papaya* | 29 |  | *Setaria italica* | 57 |
| *Lactuca sativa* | 30 |  | *Solanum pimpinellifolium* | 57 |
| *Amaranthus hypochondriacus* | 30 |  | *Oryza glumaepatula* | 57 |
| *Zostera marina* | 30 |  | *Arabidopsis thaliana* | 58 |
| *Picea abies* | 30 |  | *Mimulus guttatus* | 58 |
| *Aegilops tauschii* | 31 |  | *Petunia inflata* | 58 |
| *Ricinus communis* | 31 |  | *Solanum lycopersicum* | 58 |
| *Genlisea aurea* | 31 |  | *Capsella grandiflora* | 58 |
| *Jatropha curcas* | 32 |  | *Medicago truncatula* | 58 |
| *Morus notabilis* | 32 |  | *Brachypodium distachyon* | 59 |
| *Coffea canephora* | 33 |  | *Solanum pennellii* | 59 |
| *Ananas comosus* | 33 |  | *Vigna angularis* | 59 |
| *Vitis vinifera* | 33 |  | *Eragrostis tef* | 60 |
| *Cannabis sativa* | 34 |  | *Sorghum bicolor* | 60 |
| *Phalaenopsis equestris* | 35 |  | *Cucumis sativus* | 61 |
| *Arabidopsis lyrata* | 35 |  | *Oryza sativa subsp. japonica* | 61 |
| *Phoenix dactylifera* | 38 |  | *Phaseolus vulgaris* | 61 |
| *Oryza glaberrima* | 38 |  | *Populus euphratica* | 62 |
| *Oryza brachyantha* | 39 |  | *Triticum aestivum* | 62 |
| *Castanea mollissima* | 39 |  | *Linum usitatissimum* | 62 |
| *Dichanthelium oligosanthes* | 40 |  | *Ocimum tenuiflorum* | 63 |
| *Oropetium thomaeum* | 41 |  | *Cicer arietinum* | 63 |
| *Citrullus lanatus* | 41 |  | *Setaria viridis* | 63 |
| *Ziziphus jujuba* | 41 |  | *Catharanthus roseus* | 64 |
| *Aethionema arabicum* | 41 |  | *Panicum hallii* | 64 |
| *Nelumbo nucifera* | 41 |  | *Citrus sinensis* | 64 |
| *Arabis alpina* | 42 |  | *Sesamum indicum* | 65 |
| *Prunus mume* | 42 |  | *Actinidia chinensis* | 67 |
| *Aquilegia coerulea* | 42 |  | *Elaeis guineensis* | 74 |
| *Dianthus caryophyllus* | 43 |  | *Raphanus raphanistrum* | 74 |
| *Arachis duranensis* | 43 |  | *Solanum tuberosum* | 77 |
| *Arachis ipaensis* | 43 |  | *Zoysia pacifica* | 78 |
| *Oryza sativa subsp. indica* | 43 |  | *Malus domestica* | 80 |
| *Hordeum vulgare* | 44 |  | *Nicotiana sylvestris* | 84 |
| *Capsicum annuum* | 45 |  | *Nicotiana tomentosiformis* | 84 |
| *Dorcoceras hygrometricum* | 45 |  | *Raphanus sativus* | 85 |
| *Juglans regia* | 46 |  | *Tarenaya hassleriana* | 88 |
| *Citrus clementina* | 46 |  | *Glycine soja* | 90 |
| *Trifolium pratense* | 46 |  | *Musa acuminata* | 95 |
| *Lotus japonicus* | 47 |  | *Zea mays* | 97 |
| *Brachypodium stacei* | 47 |  | *Populus trichocarpa* | 114 |
| *Oryza barthii* | 47 |  | *Panicum virgatum* | 120 |
| *Solanum melongena* | 48 |  | *Zoysia matrella* | 121 |
| *Vigna radiata* | 49 |  | *Salix purpurea* | 124 |
| *Humulus lupulus* | 49 |  | *Brassica oleracea* | 128 |
| *Salvia miltiorrhiza* | 49 |  | *Kalanchoe laxiflora* | 139 |
| *Oryza nivara* | 49 |  | *Brassica rapa* | 139 |
| *Cucumis melo* | 49 |  | *Nicotiana tabacum* | 145 |
| *Phyllostachys heterocycla* | 51 |  | *Gossypium hirsutum* | 150 |
| *Petunia axillaris* | 51 |  | *Camelina sativa* | 153 |
| *Oryza punctata* | 51 |  | *Brassica napus* | 178 |
| *Oryza rufipogon* | 51 |  | *Glycine max* | 180 |
| *Prunus persica* | 51 |  | *Gossypium raimondii* | 181 |
| *Sisymbrium irio* | 51 |  |  |  |
