# Supplementary material for: Evolutionary Consequences of Functional and Regulatory Divergence of HD-Zip I Transcription Factors as a Source of Diversity in Protein Interaction Networks in Plants
Source: J Mol Evol. 2023 Jun 23;91(5):581–97. doi: 10.1007/s00239-023-10121-4 (PMC10598176; doi:10.1007/s00239-023-10121-4)
Supplement: Supplementary file 8 — Supplementary file8 Table S3. The number of genes within each group of the HD-Zip family in plants (DOCX 15 KB) [file 239_2023_10121_MOESM8_ESM.docx]

Table S3.

|  | HD-Zip I | HD-Zip II | HD-Zip III | HD-Zip IV |
| --- | --- | --- | --- | --- |
| *Amborella trichopoda* | 8 | 6 | 3 | 7 |
| *Arabidopsis thaliana* | 17 | 10 | 5 | 16 |
| *Brachypodium distachyon* | 12 | 12 | 4 | 14 |
| *Brassica napus* | 50 | 19 | 16 | 38 |
| *Brassica rapa* | 34 | 19 | 12 | 30 |
| *Capsicum annuum* | 21 | 9 | 10 | 15 |
| *Citrus sinensis* | 14 | 8 | 5 | 9 |
| *Cucumis sativus* | 13 | 12 | 5 | 13 |
| *Erythranthe guttata* | 14 | 10 | 8 | 21 |
| *Eucalyptus grandis* | 14 | 13 | 4 | 11 |
| *Glycine max* | 36 | 27 | 14 | 34 |
| *Gossypium hirsutum* | 57 | 35 | 28 | 29 |
| *Helianthus annuus* | 28 | 17 | 19 | 31 |
| *Hordeum vulgare subsp. vulgare* | 13 | 9 | 6 | 19 |
| *Juglans regia* | 24 | 14 | 9 | 23 |
| *Lactuca sativa* | 19 | 9 | 7 | 11 |
| *Manihot esculenta* | 22 | 16 | 8 | 13 |
| *Medicago truncatula* | 17 | 14 | 6 | 25 |
| *Musa acuminata subsp. malaccensis (banana)* | 33 | 30 | 10 | 21 |
| *Nelumbo nucifera* | 17 | 14 | 5 | 10 |
| *Nicotiana tabacum* | 46 | 25 | 11 | 29 |
| *Oryza sativa* | 14 | 14 | 7 | 12 |
| *Phoenix dactylifera* | 18 | 16 | 4 | 13 |
| *Physcomitrella patens* | 17 | 8 | 5 | 4 |
| *Populus trichocarpa* | 21 | 18 | 8 | 18 |
| *Prunus persica* | 12 | 8 | 4 | 11 |
| *Ricinus communis* | 13 | 8 | 4 | 9 |
| *Selaginella moellendorffii* | 6 | 2 | 6 | 8 |
| *Setaria italica* | 16 | 16 | 5 | 23 |
| *Solanum lycopersicum* | 24 | 11 | 6 | 45 |
| *Solanum tuberosum* | 24 | 11 | 7 | 32 |
| *Sorghum bicolor* | 13 | 12 | 5 | 22 |
| *Spinacia oleracea* | 11 | 3 | 3 | 5 |
| *Theobroma cacao* | 12 | 6 | 4 | 11 |
| *Triticum aestivum* | 33 | 39 | 15 | 36 |
| *Vitis vinifera* | 14 | 7 | 6 | 9 |
| *Zea mays* | 21 | 20 | 10 | 19 |
| *Zostera marina* | 12 | 7 | 2 | 10 |
