# Supplementary material for: Evolutionary Consequences of Functional and Regulatory Divergence of HD-Zip I Transcription Factors as a Source of Diversity in Protein Interaction Networks in Plants
Source: J Mol Evol. 2023 Jun 23;91(5):581–97. doi: 10.1007/s00239-023-10121-4 (PMC10598176; doi:10.1007/s00239-023-10121-4)
Supplement: Supplementary file 10 — Supplementary Table S5. Characterization of 17 A. thaliana HD-Zip I genes including gene and protein lengths, gene and protein organizations and biological functions (DOCX 16 KB) [file 239_2023_10121_MOESM10_ESM.docx]

Table S5.

| **Gene name** | **Arabidopsis**  **gene ID** | **CDS** | **Gene** | **Protein** | **Exon/intron** | **HD domain** | **Leucine zipper motif** | **Biological functions** |
| --- | --- | --- | --- | --- | --- | --- | --- | --- |
| ATHB1/HAT5 | At3g01470 | 819 | 2008 | 272 | 4/3 | 65-124 | 125-160 | - leaf morphogenesis  - hypocotyl elongation  - negative regulation of translation  - positive regulation of transcription  - regulation of cell growth  - response to blue light  - response to salt stress |
| ATHB3/HAT7 | At5g15150 | 945 | 2278 | 314 | 3/2 | 112-171 | 172-207 | - |
| ATHB5 | At5g65310 | 939 | 2797 | 312 | 3/2 | 69-128 | 129-164 | - positive regulation of transcription  - response to ABA |
| ATHB6 | At2g22430 | 936 | 1858 | 311 | 3/2 | 59-118 | 119-154 | - negative regulation of ABA-  activated signaling pathway  - positive regulation of transcription  - response to water deprivation |
| ATHB7 | At2g46680 | 777 | 1694 | 258 | 2/1 | 29-88 | 89-124 | - response to ABA  - ABA-activated signaling pathway  - response to water deprivation  - positive regulation of transcription |
| ATHB12 | At3g61890 | 708 | 1240 | 235 | 2/1 | 27-86 | 87-122 | - positive regulation of transcription  - response to ABA  - response to osmotic stress  - response to salt stress  - response to virus  - response to water deprivation |
| ATHB13 | At1g69780 | 885 | 1995 | 294 | 3/2 | 82-141 | 142-177 | - cotyledon morphogenesis  - leaf morphogenesis  - primary root development  - response to sucrose |
| ATHB16 | At4g40060 | 885 | 1846 | 294 | 3/2 | 56-115 | 116-151 | - negative regulation of cell growth,  photoperiodism, flowering  - positive regulation of transcription  - regulation of timing of transition from vegetative to reproductive phase  - response to blue light |
| ATHB20 | At3g01220 | 861 | 2133 | 286 | 3/2 | 84-143 | 144-179 | response to auxin |
| ATHB21 | At2g18550 | 663 | 1946 | 220 | 3/2 | 58-117 | 118-146 | - |
| ATHB22 | At2g36610 | 558 | 1080 | 185 | 3/2 | 76-135 | 136-164 | - |
| ATHB23 | At1g26960 | 768 | 1676 | 255 | 3/2 | 68-127 | 128-163 | - lateral root development  - lateral root formation  - response to gibberellin |
| ATHB40 | At4g36740 | 651 | 1989 | 216 | 3/2 | 53-114 | 112-140 | - response to auxin |
| ATHB51 | At5g03790 | 708 | 1850 | 235 | 3/2 | 74-136 | 134-162 | - bract formation  - floral meristem determinacy  - leaf morphogenesis  - positive regulation of transcription  - regulation of timing of transition from vegetative to reproductive phase |
| ATHB52 | At5g53980 | 471 | 985 | 156 | 1/0 | 8-67 | 68-96 | - response to absence of light  - response to blue light |
| ATHB53 | At5g66700 | 687 | 1611 | 228 | 2/1 | 68-127 | 128-156 | - response to auxin  - involved in root development |
| ATHB54 | At1g27045 | 684 | 1580 | 227 | 2/1 | 65-124 | 125-153 | - |
