# Supplementary material for: Evolutionary Consequences of Functional and Regulatory Divergence of HD-Zip I Transcription Factors as a Source of Diversity in Protein Interaction Networks in Plants
Source: J Mol Evol. 2023 Jun 23;91(5):581–97. doi: 10.1007/s00239-023-10121-4 (PMC10598176; doi:10.1007/s00239-023-10121-4)
Supplement: Supplementary file 11 — Supplementary Table S6. Characterization of 17 members of A. thaliana HD-Zip I subfamily including the consensus sequence of cis-regulatory element and their interacting partners. The sequence logo of cis-regulatory elements were generated by JASPAR (https://jaspar.genereg.net/) (DOCX 76 KB) [file 239_2023_10121_MOESM11_ESM.docx]

Table S6.

| **Gen name** | **AGI number** | **Sequence logo (JASPAR)** | **Interacting partners** | **Type of interaction** | **References** |
| --- | --- | --- | --- | --- | --- |
| ATHB1/HAT5 | At3g01470 | 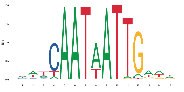 | PIF1  TBP2  AT5G12980  BRM  GRF1  AtHB1  TBP2 | Promotor  Physical  Physical  Physical  Physical  Physical  physical | BioGRID |
| ATHB3/HAT7 | At5g15150 | - | - | - | - |
| ATHB5 | At5g65310 | 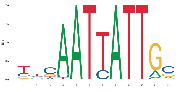 | BPM3  AUX/IAA BODENLOS (BDL)/IAA12  AT2G46260  AtHB12  AtHB7  AtHB16  AtHB6  AtHB5 | physical  Promoter  Physical  Physical  Physical  Physical  Physical  Physical | De  Smet et al. 2013 |
| ATHB6 | At2g22430 | 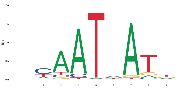 | ABI1  BPM3  BPM6  AKINBETA1  AT1G10585  AT2G28060  AT2G46260  AT3G48510  AT4G16360  AT5G48240  GRF3  GRF8  HAI1  AtHB5  KIN10  KIN11  SIP4  SNF4  SR1 | Physical  Physical  Physical  Physical  Physical  Physical  Physical  Physical  Physical  Physical  Physical  Physical  Physical  Physical  Physical  Physical  Physical  Physical  Physical | BioGRID |
| ATHB7 | At2g46680 | 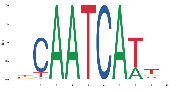 | AtTBP2, AtTFIIB  AP1  AT2G40260  AT3G19070  AT4G03250  AT4G16141  AT5G12980  CSDP1  EMB93  HB5  KELP  LCL1  MBD02  PRMT3 | Physical  Physical  Physical  Physical  Physical  Physical  Physical  Physical  Physical  Physical  Physical  Physical  Physical  Physical  Physical | BioGRID |
| ATHB12 | At3g61890 | 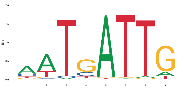 | TFIIB  AFP2  AO  AT1G10585  AT1G76210  AT2G39030  AT3G48510  AT4G16670  AT5G48430  AT5G54470  COR27  ERF8  HAI1  HB5  MGDC  MYB49  RVE2  SIP4  SR1  TMAC2  ZW9 | Physical  Physical  Physical  Physical  Physical  Physical  Physical  Physical  Physical  Physical  Physical  Physical  Physical  Physical  Physical  Physical  Physical  Physical  Physical  Physical  Physical | BioGRID |
| ATHB13 | At1g69780 | 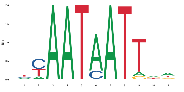 | AGL36  AT1G04850  AT1G07050  AT1G22190  AT1G69690  AT1G72740  AT2G29660  AT2G35605  AT2G42660  AT3G51470  AT3G56770  AT3G57480  AT5G05790  AT5G12980  AT5G23280  AT5G25475  AT5G44260  BZIP44  HB20  IBH1  JAZ8  MBD3  MBF1C  MYB54  SRS3  TBP2  TFIIB  TTG1  URO  WOX4  WRKY17 | Physical  Physical  Physical  Physical  Physical  Physical  Physical  Physical  Physical  Physical  Physical  Physical  Physical  Physical  Physical  Physical  Physical  Physical  Physical  Physical  Physical  Physical  Physical  Physical Physical  Physical  Physical  Physical  Physical  Physical  Physical | Ma et al. 2020  BioGRID |
| ATHB16 | At4g40060 | 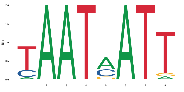 | BPM3  ERF4  HB5  MYB112 | Physical  Physical  Physical  Physical | BioGRID |
| ATHB20 | At3g01220 | 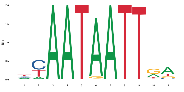 | AGL77  AT1G17310  AT1G35490  AT1G35560  AT1G48195  AT1G49475  AT1G69690  AT1G71130  AT2G42040  AT2G42660  AT2G45680  AT3G07220  AT3G10590  AT3G19070  AT3G21330  AT3G53310  AT3G57800  AT4G26030  AT4G35610  AT5G08330  AT5G12980  AT5G23280  AT5G23405  AT5G26749  AT5G51910  AT5G60142  HB13  BRM  FRS3  HB21  HB30  INO  JAZ12  MYB47  NAC020  NF-YB7  NF-YC10  NF-YC12  PTF1  RR14  RR5  SMZ  TCP10  TCP14  TCP16  TCP4  TCP5  TGA1  WRKY8 | Physical  Physical  Physical  Physical  Physical  Physical  Physical  Physical  Physical  Physical  Physical  Physical  Physical  Physical  Physical  Physical  Physical  Physical  Physical  Physical  Physical  Physical  Physical  Physical  Physical  Physical  Physical  Physical  Physical  Physical  Physical  Physical  Physical  Physical  Physical  Physical  Physical  Physical  Physical  Physical  Physical  Physical  Physical  Physical  Physical  Physical  Physical  Physical  Physical | BioGRID |
| ATHB21 | At2g18550 | 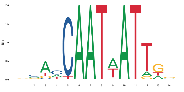 | ABF4  BRM  HB22  HB25  SWI3C  ZFHD1 | Physical  Physical  Physical  Physical  Physical  Physical | BioGRID |
| ATHB22 | At2g36610 |  | HB23  HB21  HB22  HB24  HB25  HB28  HB30  HB31  HB33  HB34  ZFHD1 | Physical  Physical  Physical  Physical  Physical  Physical  Physical  Physical  Physical  Physical  Physical | BioGRID |
| ATHB23 | At1g26960 | 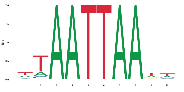 | PHYB  HB23  HB21  HB22  HB24  HB25  HB28  HB30  HB33  HB34  ZFHD1 | Physical  Physical  Physical  Physical  Physical  Physical  Physical  Physical  Physical  Physical  Physical | BioGRID |
| ATHB40 | At4g36740 | 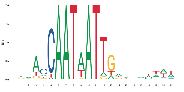 | BRM  SWI3B | Physical  Physical | BioGRID |
| ATHB51 | At5g03790 | 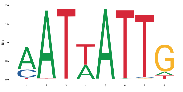 | CAL  ARR11  AT1G13450  AT1G17310  AT1G48150  AT2G01818  AT5G41030  CO  DA1  RAP2.9 | Promoter  Physical  Physical  Physical  Physical  Physical  Physical  Physical  Physical  Physical | BioGRID |
| ATHB52 | At5g53980 | - | - | - | - |
| ATHB53 | At5g66700 | 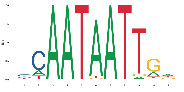 | ADA2A  AT4G12850  AT5G12980  BRM  LBD21  SWI3C | Physical  Physical  Physical  Physical  Physical  Physical | BioGRID |
| ATHB54 | At1g27050 | - | - | - | - |

References

De Smet R, Adams KL, Vandepoele K et al (2013) Convergent gene loss following gene and genome duplications creates single-copy families in flowering plants. Proc Natl Acad Sci USA. 110:2898-903. <https://doi.org/10.1073/pnas.1300127110>

Ma YJ, Li PT, Sun LM, Zhou H et al (2020) HD-ZIP I transcription factor (*PtHB13*) negatively regulates citrus flowering through binding to FLOWERING LOCUS C promoter. Plants (Basel). 9:114.<https://doi.org/10.3390/plants9010114>
